# Controlling the Collective Transport of Large Passive Particles With Suspensions of Microorganisms

**DOI:** 10.1002/smll.202511502

**Published:** 2026-02-27

**Authors:** Taha Laroussi, Julien Bouvard, Etienne Jambon‐Puillet, Mojtaba Jarrahi, Gabriel Amselem

**Affiliations:** ^1^ Laboratoire d'Hydrodynamique (LadHyX), CNRS, Ecole Polytechnique Institut Polytechnique de Paris Palaiseau France; ^2^ Living Systems Institute University of Exeter Exeter United Kingdom; ^3^ Université Paris‐Saclay, CNRS, FAST Orsay France

**Keywords:** active matter, bioconvection, cargo delivery, decontamination, microswimmers, transport

## Abstract

A promising approach to transport cargo at the microscale lies within the use of self‐propelled microorganisms, whose motion entrains that of passive particles. However, most applications remain limited to just a few passive particles of similar size as the microorganisms, since the transport mechanism relies on the interaction between individual swimmers and single particles. Here, we demonstrate how to control the collective transport of hundreds of large passive particles with phototactic microalgae. Using directional light stimuli in suspensions of *Chlamydomonas reinhardtii*, we trigger bioconvection rolls capable of macroscale transport. Passive particles an order of magnitude larger than the microalgae are either attracted or repelled by the rolls, depending on their density. Using experiments and simulations, we rationalize these bioconvective flows and describe how to harness them for cargo transport, with future applications in targeted drug delivery and decontamination.

## Graphical abstract

Directional light stimuli induce the accumulation of microalgae in suspensions of *Chlamydomonas reinhardtii*. This accumulation triggers large scale bioconvection rolls capable of macroscale transport. Exploiting these bioconvective flows enables to collectively transport of hundreds of large passive particles, paving the way for future applications in decontamination and drug delivery using phototactic microalgae.

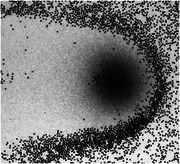

## 1 Introduction

Despite several applications with high societal impact, such as soil and ocean remediation [1] or targeted drug delivery [2, 3, 4], controlled cargo transport remains a challenge at the submillimetric scale. These different applications have distinct goals and constraints, which prevents a “one‐size fits all” solution. For instance, targeted drug delivery requires the precise transport of a relatively small cargo. For remediation purposes, however, it may be more desirable to quickly sweep away pollutants, such as microplastics, from a large area to a collection point but without much precision.

Harnessing the motility of self‐propelled microorganisms is a promising approach to control the transport of a passive cargo at the microscale. One method is to physically bind cargo, e.g. a bead, to the surface of motile cells, such as amoeba [5], algae [6] or bacteria [7, 8]. These beads are then carried by the cells as they crawl or swim. Control can be achieved by directing the cell motion with external cues to which cells respond, such as chemical gradients or light stimuli. Another strategy is to use the flows created by swimming microorganisms to displace suspended particles. These flows usually extend over a distance of roughly one microorganism (≈1−10μm), and can entrain neighboring colloids [9]. When confined inside chambers with specific branched geometries, the interaction between microalgae and passive particles can lead to demixing, with colloids eventually accumulating in certain regions of the device, which could facilitate their collection for decontamination applications [10].

However, all these transport mechanisms rely on the interaction between a single microorganism and a single passive particle, usually smaller than the microorganism itself. At larger scale, passive beads mixed in a suspension of active fluids such as swimming bacteria or microalgae move randomly, albeit with an effective diffusion coefficient several orders of magnitude larger than predictions from the traditional Stokes–Einstein relationship [11, 12, 13, 14, 15, 16]. Increasing the concentration of passive particles, one can eventually observe segregation, with the formation of clusters ranging in size from as few as ten beads [17, 18], to very large clusters up to $10^{5}$ particles [13]. Yet, this large‐scale clustering stems from random motions and cannot be easily controlled. To obtain a directional motion, one needs to break the system symmetry, which has previously been achieved by designing geometrically asymmetric structures that guide the swimmer's movement [10, 19, 20, 21, 22, 23, 24]. However, this method requires careful manufacturing, and is not adaptable. The direction has to be planned in advance and a specific microdevice needs to be prepared. Would it be possible to transport a large number of particles, without size constraints, and control the transport direction dynamically?

Here, we leverage collective effects in suspensions of the swimming microalgae *Chlamydomonas reinhardtii* to achieve collective directional transport at the microscale. Rather than relying on surface patterning to direct the microswimmer's motion, we use a directional light stimulus, which can be tuned dynamically. By locally accumulating microalgae which are denser than water, we trigger macroscale bioconvection patterns [25, 26, 27, 28, 29] that we harness to transport particles. Beads from 50 to 460μm, up to 50 times larger than a single microalga, are successfully transported collectively over several millimeters along a prescribed light path. We propose a simple continuous description of the algae suspension that we solve numerically to predict the flow profile and the bead motion, and find good agreement with experiments. Depending on the buoyancy of the beads, these bioconvection rolls can either direct particles to a target location or sweep them away from a given area, highlighting the versatility and potential of this system for applications.

## 2 Results

### 2.1 Beads Move Collectively in Response to Light Stimuli

**FIGURE 1:**
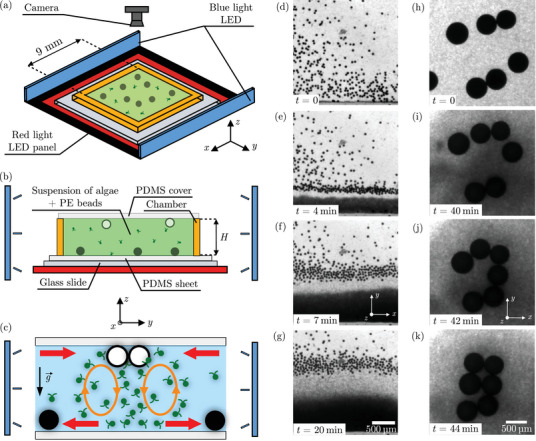
Experimental setup ‐ Photo‐bioconvective flows displace large beads. (a) Schematic image of the setup. A square chamber of width 9mm and height H is placed on top of a red light LED panel. The experiment is recorded with a camera placed above the chamber. Two blue light LED bands are placed on each side of the chamber. (b) Cross‐sectional view of the chamber. The chamber of height H is filled with a suspension of algae *Chlamydomonas reinhardtii* and polyethylene (PE) beads. (c) Sketch of the recirculating convection rolls that appear when algae accumulate in a region of the chamber. Passive particles in the range of those rolls are either attracted or repelled depending on their relative density with the medium. Dense beads are drawn in black and light beads in white. (d–g) Repulsion of denser beads of diameter $d_{b}=50\;\mu \;m$, located on the bottom surface, in a chamber of height H=310μm (top view). As negatively phototactic algae accumulate at the lateral wall, beads denser than the fluid are pushed away, forming a front (see Movie S1). (h–k) Attraction of lighter beads of diameter $d_{b}=460\;\mu \;m$, located on the top surface, in a chamber of height H=930μm (top view). Both lateral LEDs are then switched on, forming a region of highly concentrated algae which draws in beads lighter than the fluid (see Movie S2). Both experiments (d–k) are conducted with an initial optical density ${\mathrm{OD}}_{i}$ of 10, i.e. $c_{i}=3\times 10^{7}\;\mathrm{cells}\;{\mathrm{mL}}^{-1}$.

Experiments begin by enclosing a suspension containing submillimetric polyethylene microspheres, of diameter $d_{b}=50\;\mu \;m$ and median density $\rho_{b}\approx 1006\;\mathrm{kg}\;m^{-3}$ (see Methods), with microalgae *Chlamydomonas reinhardtii* in a square rectangular chamber of side 9 mm and height H=310μm. The chamber is placed on top of a red LED panel (λ=630nm) for visualization purposes, and imaged from above. Two blue LED strips (λ=470nm) are positioned on opposite sides of the chamber to stimulate the algae, see sketches in Figure 1a,b and Materials and Methods for more details. Initially, the blue LEDs are off, and the beads and algae are homogeneously distributed throughout the chamber, see Figure S1b. Unless noted otherwise, the algal suspension contains $c=3\times 10^{7}\;\mathrm{cells}\;{\mathrm{mL}}^{-1}$, corresponding to an optical density OD=10.

When one LED strip is switched on, the algae respond by negative phototaxis and swim away from the intense blue light [30, 31, 32]. This leads to all algae accumulating at the edge of the chamber opposite to the light source, see time‐lapse in Figure 1d–g. At the same time, the microspheres suspended with the algae dramatically reorganize: while initially evenly distributed throughout the chamber, these beads, five times larger than an individual microalga, are collectively pushed away from the dense algal zone and form a front distinctly separated from the dense algal region, see time‐lapse in Figure 1d–g and Movie S1.

Repeating the exact same experiment with beads of density $\rho_{b}\approx 990$ kg $m^{-3}$ and diameter $d_{b}=460\;\mu \;m$ leads to the opposite behavior: the zone dense in algae attracts nearby beads, leading to the formation of a raft of beads, see time‐lapse in Figure 1h–k, Movie S2 and Figure S7. Note that the beads in this experiment are almost 50 times larger than a single microalgae. On a longer time scale (∼1 h), these floating beads can “surf” over the concentrated algal regions, hopping on and off as they develop and disappear, see Figure S8.

Experiments with beads of density either 990 or 1006 kg $m^{-3}$ and diameters ranging between $d_{b}=50$ and 460μm reveal that the collective motion of beads occurs systematically, for all bead diameters. Its direction depends solely on the bead density: dense beads are pushed away from the zone concentrated in algae, while buoyant beads are attracted toward it. This suggests the existence of a convective flow, going away from the dense region of algae at the bottom of the chamber and toward the algae at the top of the chamber, see sketch in Figure 1c.

As shown in refs. [25, 27], concentrating algae with light in localized regions can create buoyancy driven flows. Since algae are denser than water with a density $\rho_{a}\approx 1050$ kg $m^{-3}$ [25, 27], a lateral concentration gradient of algae therefore leads to a lateral density gradient (perpendicular to gravity). This situation is unstable [33, 34], and triggers a flow in which the denser region (algae‐rich) falls and flows toward the lighter region (algae‐poor), which itself rises and flows toward the denser region.

In the absence of forcing, this flow is transient since it mixes the fluid and thus erases the density gradient. However, in our case it is sustained as long as the light stimulus is on. Indeed, while algae are advected away from the dense region by the buoyancy driven flow, their negative phototaxis makes them swim back toward the dense region, away from the strong light. This repopulates the dense algal region [25] which reaches a steady‐state, see sketch in Figure 1c. Note that, unlike the well‐known Rayleigh‐Bénard instability where temperature differences generate a density gradient parallel to gravity, this convective flow due to a lateral density gradient occurs without any threshold [33, 34].

The bioconvection rolls can be experimentally observed by following beads with a density ≈1001 kg $m^{-3}$ close to the surrounding medium's density. At first, these beads are pushed away from the region of high algal density. After traveling a few hundred microns, they enter upwelling flows, reaching the top lid before recirculating toward the side wall, eventually returning to the algae‐dense region, see Figure S6a. Some beads can even stay inside the convection rolls for up to 1 h, following the recirculating flows. Looking from the top, these beads seem to be “bouncing” on the edge of the dense algal region, see Figure S6b and Movie S3. Such bead trajectories highlight the simultaneous presence of inward (attractive) and outward (repulsive) flows at different heights within the chamber.

### 2.2 A Minimal Numerical Continuous Model of Photo‐Bioconvection Yields Quantitative Predictions of Bead Trajectories

To quantitatively understand these photo‐bioconvection rolls and the transport of beads by the algae, we develop a continuous mathematical model of the suspension of algae. Bioconvection can be rationalized by idealizing algae as an active concentration field c coupled to the Navier–Stokes equation describing the fluid velocity u [35]. Coarse‐graining the microorganism microscopic motion results in an advection‐diffusion equation for c with an effective diffusion coefficient D representing random motion, and a self‐advection velocity representing directed motion due to any of the ‘taxis’ (chemotaxis, gravitaxis, phototaxis, rheotaxis...). Coupling with the fluid flow occurs through the advection of c but can also appear in the ‘taxis’ (e.g. gyrotaxis or rheotaxis), through the mixture density, or in fluid stresses that can incorporate cell local stresses in addition to viscous stresses.

In an attempt to gain physical insights into the phototactic bioconvection rolls observed in our experiments, we keep the model as simple as possible. The experiment being invariant in the x direction (see Figure 1), we only consider a 2D (y,z) slice of the chamber and incorporate only key physical ingredients: phototaxis with an advection velocity $u_{\mathrm{photo}}$ and density coupling $\rho \left(c\right)=\rho_{w}+\phi_{a}\left(\rho_{a}-\rho_{w}\right)$ with the Boussinesq approximation. Here, $\rho_{w}$ is the fluid density in the absence of algae, similar to the fluid density of water, $\rho_{a}$ is the density of algae, and $\phi_{a}=\left(4/3\right)\pi R_{a}^{3}c$ is the algal volume fraction, estimated assuming an individual alga is a sphere of radius $R_{a}≃5\;\mu \;m$ [27]. As previously done for phototactic *C. reinhardtii* [25, 27], we thus neglect gravitaxis, gyrotaxis, cell stresses, and assume all algae behave identically. The coupled equations for the concentration c(y,z,t) and fluid velocity u(y,z,t) are:

(1) $$\begin{array}{cc} \rho_{w}\left(\frac{\partial u}{\partial t}+\left(u\cdot \nabla \right)u\right) & =-\nabla p+\eta \Delta u+\rho (c)g \end{array}$$

(2) $$\begin{array}{cc} \nabla \cdot u & =0 \end{array}$$

(3) $$\begin{array}{cc} \frac{\partial c}{\partial t}+\nabla \cdot \left[(u+u_{\mathrm{photo}})c\right] & =D\Delta c \end{array}$$

Here, p(y,z,t) is the pressure, η is the fluid viscosity, and g is gravity.

We observe that algae swim away from the light and notice that they form a band of constant concentration $c_{\max}\approx 3\times 10^{8}$ cells ${\mathrm{mL}}^{-1}$ at the lateral wall (see Figure 1e), corresponding to a volume fraction $\phi_{a,\max}$< 25 %, hinting that some physical processes that we have neglected prevent them from becoming more concentrated. These could include finite size interactions, local fluid stresses, or light shielding. For simplicity, we thus assume $u_{\mathrm{photo}}=-u_{\mathrm{photo}}\left(1-c/c_{\max}\right)e_{y}$. This phototactic velocity simplifies to a constant velocity $-u_{\mathrm{photo}}e_{y}$ in the dilute regime, while accounting for crowding effects when concentration increases.

We solve Equations (1)–(3) numerically in a rectangular domain of the experimental chamber dimensions (9mm×310μm) with the finite element solver COMSOL. We use a no flux boundary condition for the concentration c, a no slip boundary condition for the velocity u, and assume a constant initial algae concentration $c\left(y,z,0\right)=c_{i}$ and zero initial velocity u(y,z,0)=0. In practice, we solve Equation (3) for the rescaled concentration $\bar{c}=c/c_{\max}$ that varies between 0 and 1 and use $\rho \left(\bar{c}\right)=\rho_{w}+\bar{c}\left(\rho_{\max}-\rho_{w}\right)$ in Equation (1) (see Methods). All model parameters apart from the velocity $u_{\mathrm{photo}}$ can be measured experimentally. The diffusion coefficient $D=4\times 10^{-9}$ $m^{2}$ $s^{-1}$ is measured independently (see Section SI.C), $\rho_{\max}\approx$ 1008 kg $m^{-3}$ and $\rho_{w}\approx$ 1000 kg $m^{-3}$ are extracted from experimental images for each experiment, and $\eta =1\times 10^{-3}$ Pa s. The average swimming velocity of *C. reinhardtii* is measured as 40–60 $\mu {m\;s}^{-1}$ under a microscope (see Figure S4). All phototactic speeds tested in the range $u_{\mathrm{photo}}∼$ 1–100 $\mu {m\;s}^{-1}$ systematically lead to an accumulation of algae at the edge of the simulation box, and a self‐sustained convective roll. Adding gravitaxis to account for the bottom heaviness of the algae [36] or taking a crowding term of the form ${\left(1-c/c_{\max}\right)}^{n}$ with n≠1 has only a small influence on the steady‐state convective roll characteristics (see Figure S9 and Section SII.B.).

**FIGURE 2:**
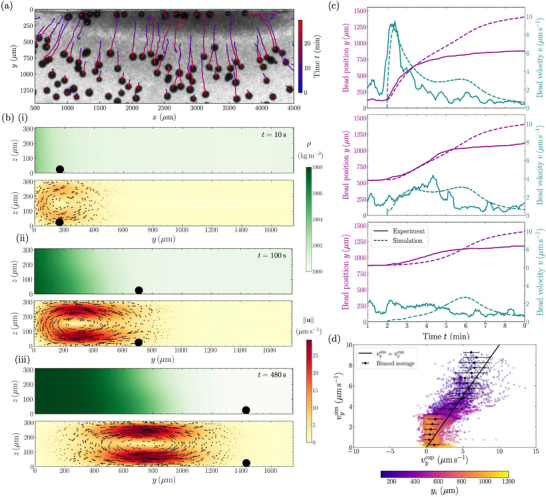
Bead dynamics within bioconvective flows. (a) Experimental trajectories of beads of diameter $d_{b}=110\;\mu \;m$, in a chamber of height H=310μm, pushed away from the chamber lateral wall by phototactic *C. reinhardtii* accumulating at the top boundary, ${\mathrm{OD}}_{i}=10$ (top view). (b) Simulated evolution of the local density ρ in the chamber, alongside the flow field it generates. Red regions correspond to zones of high velocity and illustrate the formation and outward propagation of bioconvective rolls. The successive positions of a simulated bead of diameter $d_{b}=50\;\mu \;m$ sitting on the floor of the chamber is indicated by black disks. (i): t=10s. (ii): t=100s. (iii): t=480s. See Movie S4. (c) Position y normal to the wall (magenta), and velocity v (teal) of three representative beads of diameter $d_{b}=50\;\mu \;m$, initially located at increasing distances from the wall. Experimental data are shown as solid lines while simulated data with a $t_{\mathrm{lag}}≃2$ min are displayed as dashed lines (blue‐light is switched on at t=0). Top: A bead, initially close to the wall ($y_{i}=125\;\mu \;m$), is rapidly accelerated by the algae‐induced bioconvective flow, reaching a peak velocity of approximately $10\;\mu m\;s^{-1}$, before decelerating and entering a steady regime at $0.5\;\mu m\;s^{-1}\;\;\mathrm{to}\;\;1\;\mu m\;s^{-1}$. Experiment and simulations are in excellent agreement. Middle: A bead starting farther away ($y_{i}=545\;\mu \;m$) is accelerated more gradually and reaches a lower peak velocity. Experiment and simulations are in very good agreement. Bottom: A bead initially far from the wall ($y_{i}=880\;\mu \;m$) moves at much lower speeds. Experiment and simulations are in good agreement (see main text for discussion). (d) Instantaneous simulated bead velocity $v_{y}^{\mathrm{sim}}$ as a function of the instantaneous experimental velocity $v_{y}^{\exp}$, for beads initially located within 1200μm of the wall for an initial optical density ${\mathrm{OD}}_{i}=10$. Their initial position $y_{i}$ is color‐coded from purple ($y_{i}=107\;\mu m$) to yellow ($y_{i}=1200\;\mu m$). The black line shows $v_{y}^{\mathrm{sim}}=v_{y}^{\exp}$.

To be more quantitative, we focus on dense ($\rho_{b}\approx$ 1006 kg $m^{-3}$) beads of diameter $d_{b}=50\;\mu \;m$ in an algal suspension at concentration 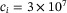 cells ${\mathrm{mL}}^{-1}$. A typical bead displacement tracking is shown in Figure 2a (see Supporting Information for details). To compare experimental results to simulations, we measure the initial algae concentration $c_{i}\left(y\right)$ along the y direction, at t=0 before the blue‐light is turned on, and use it as the initial condition in the simulations. We take an advection velocity $u_{\mathrm{photo}}=35\;\mu {m\;s}^{-1}$, which matches the experimental time required for the algae to accumulate on one side of the chamber. We then obtain the numerical density/concentration profile and fluid velocities shown in Figure 2b and Movie S4. Initially, the algae concentration is small and almost uniform. As the algae swim to the left and concentrate near the lateral wall, a weak convection roll immediately forms at the wall. This convection roll becomes stronger and wider as more algae accumulate, with fluid velocities reaching up to ||u||≈ 25 $\mu {m\;s}^{-1}$. Eventually, the concentration at the wall reaches $c\approx c_{\max}$. After this point, more algae accumulation creates a growing band at $c\approx c_{\max}$ near the wall, and the concentration front between the algae‐rich and poor regions moves away from the wall. The convection roll moves with the front while keeping the same velocity magnitude and width, see Movie S4 for a more detailed view of the roll dynamics. The beads velocities and trajectories are calculated assuming they behave as passive tracers (see Methods) and shown alongside experiments in Figure 2c for three representative beads (dashed lines).

In the experiment, we observe that the beads start to move roughly two minutes after the light is turned on at t=0, whereas in the numerical model motion is instantaneous. Correcting for this lag $t_{\mathrm{lag}}$ not captured by the model, we find very good agreement between simulations and experimental results, with both the correct shape for the time evolution of the bead velocity and a correct order of magnitude, which depends on the initial bead position. The motion of beads is better predicted for beads initially closer to the lateral wall, that are initially taken up in the convection roll and are advected away from the forming dense algal region at speeds up to $\approx \;\;\;\;10\;\mu \;m\;\min^{-1}$ (see Figure 2c, top). This phase of motion lasts ≈1min, during which beads can travel up to ≈500μm, see magenta line in Figure 2c, top, for 2≤t≤3min. Then, these beads exit the core of the convection roll. They continue to be pushed away from the dense algal region, albeit at a much slower characteristic speed, of order $\approx \;\;\;\;0.5\;\mu \;m\;\min^{-1}$. In contrast, beads initially far away from the wall never enter the core of the convection roll. Their experimental and simulated velocities are smaller than $\approx \;\;\;\;4\;\mu \;m\;\min^{-1}$, see Figure 2c middle and bottom. The simulations slightly overestimate these beads velocity, likely because we have neglected the bead friction with the wall. Nevertheless, these beads also exhibit a similar slow speed of order $\approx \;\;\;\;0.5\;\mu \;m\;\min^{-1}$ at longer times, t≥8min, while pushed away from the roll.

Now taking into account all tracked beads, we plot in Figure 2d the experimental instantaneous velocity against its simulated value for each bead at each time. We find again a good agreement between simulations and experiments, notably for bead velocities $v_{y}^{\exp}≳3\;\mu \;m\;s^{-1}$, corresponding to beads initially within 400μm of the channel wall, see Figure 2d. Simulations overestimate bead velocities when experimental bead velocities are small ($v_{y}^{\exp}≲3\;\mu \;m\;s^{-1}$), which occurs for beads further away than 400μm from the wall initially. In particular, for beads with experimental velocities around $v_{y}^{\exp}\approx \;\;1\;\mu \;m\;s^{-1}$, the corresponding simulated velocities reach up to $v_{y}^{\mathrm{sim}}\approx \;\;4\;\mu \;m\;s^{-1}$. This hints again at effects slowing down the beads experimentally, such as lubrication or friction. The study of these second‐order effects is left for future work. All individual bead velocity profiles, simulated and experimental, are shown in Figure S10.

### 2.3 Collective Bead Motion is Driven by the Dynamically Evolving Roll of Bioconvection

Armed with our understanding of the motion of single particles, we now consider the motion of a collection of beads. Experiments show that dense beads accumulate along a line, away from the dense algal region, see pictures in Figure 1f,g. This line will hereafter be referred to as the front of beads, see Figure S5 for details on its definition and detection. Its dynamics, which effectively corresponds to the collective motion of hundreds of individual particles, is studied and quantified to understand its origin. In particular, at long times t≳10min, what sets the position of this front of beads?

**FIGURE 3:**
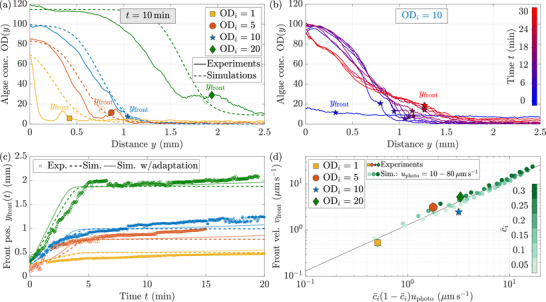
Denser beads form a front when repelled from dense algal regions. (a) Profiles of algal concentration OD(y) near the lateral wall (y=0) for different initial optical densities ${\mathrm{OD}}_{i}$. The profiles are taken t=10min after the blue LED is switched on. The position of the front of beads $y_{\mathrm{front}}$ is denoted for each curve with a filled colored symbol. Simulated profiles, in dashed lines, are also superimposed with their respective front position which is calculated as the position where the vertical fluid velocity is maximal (hollow symbols). (b) Temporal evolution of the experimental algal concentration profile OD(y) near the wall (y=0) for an initial optical density ${\mathrm{OD}}_{i}=10$. Initially, there is no light stimulus so the profile is roughly flat. After the blue LED is switched on at t=0, the algae begin to accumulate at the wall. The position of the beads front $y_{\mathrm{front}}$ is denoted at each time with a colored star. See Figure S13 for a comparison between the experimental and simulated profiles. (c) Temporal evolution of the position of the beads front $y_{\mathrm{front}}$ for different initial optical densities ${\mathrm{OD}}_{i}$, in the experiments (symbols) and in the numerical simulations without (dashed lines) or with adaptation (dotted lines). (d) The front velocity $v_{\mathrm{front}}$ scales linearly with $u_{\mathrm{photo}}{\bar{c}}_{i}\left(1-{\bar{c}}_{i}\right)$, both in the simulations (small symbols) and in the experiments (large symbols with errorbars). Simulations were run with a normalized initial concentration ${\bar{c}}_{i}=c_{i}/c_{\max}$ ranging from 0.05 to 0.3, corresponding to $c_{i}=1.1\times 10^{7}-1.0\times 10^{8}\;{\mathrm{cells}\;\mathrm{mL}}^{-1}$ or ${\mathrm{OD}}_{i}=3.8\;\;\;\mathrm{to}\;\;34$, and a phototactic velocity $u_{\mathrm{photo}}=10\;\mu \;m\;s^{-1}\;\;\mathrm{to}\;\;80\;\mu \;m\;s^{-1}$. Solid line: $v_{\mathrm{front}}=1.3\;{\bar{c}}_{i}\left(1-{\bar{c}}_{i}\right)u_{\mathrm{photo}}$.

The initial cell seeding concentration is varied from $3\times 10^{6}\;{\mathrm{cells}\;\mathrm{mL}}^{-1}$ to $6\times 10^{7}\;{\mathrm{cells}\;\mathrm{mL}}^{-1}$, respectively corresponding to an optical density of 1 and 20, and the cells are subjected to the same directional light stimulus in all experiments. In all cases, algae accumulate away from the light source, triggering bioconvection. Dense 50μm beads are then pushed away from the concentrated region in algae. The local algal concentration at all locations in the chamber is retrieved from the experimental images at all times, using the pixels' gray level intensity, see Figure S4. The algal concentration profile 10 min after the onset of stimulation is shown in Figure 3a as solid lines, for different initial cell seeding concentrations. As expected, the higher the initial concentration of algae, the larger the accumulation region. In each case, the position of the front of beads $y_{\mathrm{front}}$, superimposed on the concentration profiles as filled symbols in Figure 3a, is systematically several hundreds of microns away from the dense algal region.

For an initial optical density ${\mathrm{OD}}_{i}=10$, the temporal evolution of the algal concentration profile is shown in Figure 3b. The near‐uniform concentration profile at t≤0, before the lighting of the blue LED, becomes peaked close to the lateral wall within minutes. Then, a stationary profile of algal concentration emerges, reflecting an equilibrium between the negative phototactic behavior of the algae, which makes them swim away from the light, i.e. toward the wall at y=0, and both diffusion and convection, which tend to homogenize the concentration. At ${\mathrm{OD}}_{i}=10$, the stationary state is reached in about 6 min with the algae accumulation spanning from y=0 to 1 mm. The front of beads, superimposed on the profiles as colored stars, is moving away from the dense concentrations appearing close to the wall, staying at the edge of the convection rolls in a region where the local concentration OD(y) does not exceed 20. For t≥10min, the concentration profile slowly flattens, resulting in a slower advance of the beads front after the initial burst of speed.

The evolution of the position of the front of beads $y_{\mathrm{front}}\left(t\right)$ with time is shown for four initial cell seeding concentrations, from ${\mathrm{OD}}_{i}=1$ to ${\mathrm{OD}}_{i}=20$, in Figure 3c. In all cases, the front displays an initial faster phase of motion during ≈5min, with a quasi‐constant speed of the order of a couple $\mu \;m\;\min^{-1}$, after which a slower front displacement is observed, with a speed of the order of $\approx \;\;\;\;0.1\;\mu \;m\;\min^{-1}$. These two phases of motion are all the clearer for higher values of the initial algal concentration ${\mathrm{OD}}_{i}$.

Experiments are then compared to the numerical solution of Equations (1)–(3), using as initial conditions a quiescent fluid and a concentration profile of algae identical to the experimental algae concentration profile at t=0 when the blue LED is switched on. A bioconvection roll forms in the simulation, and the front position in the simulations is defined as the position where the vertical fluid velocity is maximal, corresponding to the extremity of the convection roll. We find very good agreement up to t≈10min between experiments and simulations, see the dashed lines and symbols in Figure 3a for the simulated concentration profiles and front position at t=10min, respectively, and dashed lines in Figure 3c for the simulated front dynamics.

A front velocity $v_{\mathrm{front}}$ can be measured from the initial faster phase of motion of the front of beads (t≤5min). This velocity is quasi‐constant, and can be simply defined as $v_{\mathrm{front}}^{\exp}=\left(y_{\mathrm{front}}\left(t\right)-y_{\mathrm{front}}\left(t_{\mathrm{ref}}\right)\right)/\left(t-t_{\mathrm{ref}}\right)$, for all values of t such that $t_{\mathrm{ref}}<t\leq 5\;\min$. The reference time $t_{\mathrm{ref}}$ is chosen as the first time the front can be reliably detected; we experimentally find $t_{\mathrm{ref}}=1.5\;\min$ for ${\mathrm{OD}}_{i}=1$ and $t_{\mathrm{ref}}=0$ for all other initial concentrations. The front velocity is then calculated for t=2,3 and 4min, with its average value being plotted in Figure 3d as large symbols and its standard deviation as error bars. To compare with numerical simulations, we assimilate $v_{\mathrm{front}}$ with the advance speed of the convection roll, which is also constant over the first phase of motion, see Figure 3c. Velocities from the simulations are computed for different normalized initial seeding concentrations ${\bar{c}}_{i}=0.05\;\;\;\mathrm{to}\;\;0.3$, corresponding to $c_{i}=1.1\times 10^{7}-1.0\times 10^{8}\;{\mathrm{cells}\;\mathrm{mL}}^{-1}$ or ${\mathrm{OD}}_{i}=3.8\;\;\;\mathrm{to}\;\;34$, and different phototactic velocities $u_{\mathrm{photo}}=10\;\mu \;m\;s^{-1}\;\;\mathrm{to}\;\;80\;\mu \;m\;s^{-1}$. We find that the front velocity increases linearly with the product ${\bar{c}}_{i}\left(1-{\bar{c}}_{i}\right)u_{\mathrm{photo}}$, as highlighted by the small filled circles in Figure 3d. Experimental data on the front velocity are in excellent agreement with simulations, see large symbols in Figure 3d.

The front velocity $v_{\mathrm{front}}$ at early times (t≲5min) can be retrieved using the conservation of the number of algae. Indeed, the number of algae in the concentrated algal region is $n\left(t\right)\approx c_{\max}Hw\left(t\right)$, where w(t) is the width of the concentrated region of algae at time t. The flux of algae into this region is due to algae from the bulk swimming toward the concentrated region: $dn/dt\approx c_{i}\left(1-c_{i}/c_{\max}\right)Hu_{\mathrm{photo}}$, where $c_{i}$ is the algal concentration in the bulk. We therefore find

(4) $$\frac{dw}{dt}\approx \frac{c_{i}}{c_{\max}}\left(1-\frac{c_{i}}{c_{\max}}\right)u_{\mathrm{photo}}={\bar{c}}_{i}\left(1-{\bar{c}}_{i}\right)u_{\mathrm{photo}}$$

with ${\bar{c}}_{i}=c_{i}/c_{\max}$, in excellent agreement with numerical simulations and experiments for t≲10min, see solid line in Figure 3d.

Yet, longer time dynamics are not well captured by the model: the simulated front stops moving after t≈5min, see dashed lines in Figure 3c, while the experimental front of beads continues moving. This discrepancy can be understood by monitoring in experiments the time evolution of the algal concentration profile. As the algae concentration builds up at initial times (t<6min), the front of beads moves over ≈1mm. Then, after t≈12min of light stimulation, the algae concentration profile flattens, see red lines in Figure 3b. While early times are well captured by Equations (1)–(3), the flattening of the algae concentration profile, which is striking without beads clouding the concentration profiles (see Figure S12), is not reproduced by the model, as highlighted in Figure S13a,b. Indeed, this latter evolution is due to the adaptation of *C. reinhardtii* to the light stimulus, a well‐documented phenomenon [26, 37, 38]. In response to a sustained strong light stimulus, algae initially experience negative phototaxis and swim away from the light; after a dozen minutes, they adapt to the light level and decrease their avoidance behavior, sometimes even switching to positive phototaxis after longer times, on the order of dozens of minutes to an hour [26, 37]. At the population level, adaptation corresponds to a mean phototactic velocity that continuously decreases, and can even change sign at long times.

To take into account adaptation in the model, the swimming speed is imposed to be a decreasing function of time, $u_{\mathrm{photo}}\left(t\right)=\left(u_{\mathrm{photo}}\left(0\right)-\alpha t\right)$, with $u_{\mathrm{photo}}\left(0\right)=43\;\mu \;m\;s^{-1}$ and $\alpha =0.019\;\mu \;m\;s^{-2}$. We then recover excellent agreement between simulated and experimental concentration profiles at all times (see Figure S13c), as well as between the simulated and experimental front dynamics, see dotted lines in Figure 3c. In particular, we retrieve the sustained slow motion of the front of beads at times larger than t≳10min, due to the expansion of the convection roll.

### 2.4 Collective Particle Motion: Applications to Surface Cleaning and Directional Transport

In configurations where beads are denser than the surrounding fluid and sediment, convective flows can be used to sweep the bottom surface of the environment. To highlight the potential of photo‐bioconvective flows in surface cleaning applications, we define three quantitative performance metrics that we apply to the controlled experiments analyzed in Figure 3. The first metric, the bead surface fraction $\Psi_{b}$, quantifies how many beads are swept away from a predefined region of interest; the cleaning efficiency ε measures how many beads are excluded from the dense algae region; last, the total cleaned surface $S_{\mathrm{clean}}$ assesses the extent of the region exempt of beads (“cleaned”) in the entire chamber.

**FIGURE 4:**
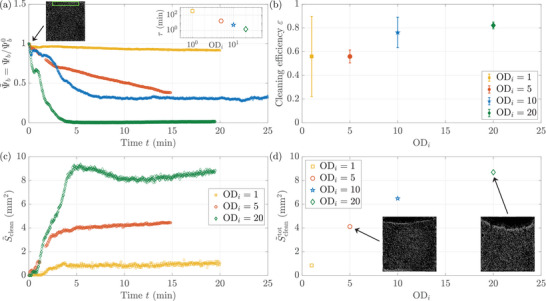
Surface cleaning metrics. (a) Temporal evolution of the bead surface fraction on the bottom surface $\Psi_{b}$, normalized by its initial value $\Psi_{b}^{0}=\Psi_{b}\left(t=0\right)$ before the blue light is switched on. $\Psi_{b}$ is measured in a 6×0.9mm region, corresponding to 67×10% of the whole chamber, located at the wall opposite of the light stimulus (see green rectangle in left inset, a typical image of beads in the entire 9×9mm chamber at t=0). Right inset: Characteristic cleaning time τ, calculated from the exponential fits ${\tilde{\Psi }}_{b}={\tilde{\Psi }}_{0}\exp\left(\left(t_{0}-t\right)/\tau \right)$. (b) Cleaning efficiency ε of the beads by the algal bioconvection rolls, as a function of the initial optical density ${\mathrm{OD}}_{i}$. ε=1 means that every bead was successfully swept away. ε is measured by comparing the bead surface fraction, in the region located between the wall and 200μm ahead of the final bead front position, between t=0 and after the roll has vanished. (c) Temporal evolution of the cleaned surface ${\tilde{S}}_{\mathrm{clean}}\left(t\right)$ across the whole chamber. ${\tilde{S}}_{\mathrm{clean}}\left(t\right)$ corresponds to $S_{\mathrm{clean}}$ subtracted by the initial value $S_{\mathrm{clean}}^{0}=S_{\mathrm{clean}}\left(t=0\right)$ before the blue light is switched on. A region is considered cleaned if there are strictly less than 2 beads in a 200×200μm region. (d) Total cleaned surface ${\tilde{S}}_{\mathrm{clean}}^{\mathrm{tot}}$ across the entire chamber, as a function of the initial optical density ${\mathrm{OD}}_{i}$. ${\tilde{S}}_{\mathrm{clean}}^{\mathrm{tot}}={\left\langle {\tilde{S}}_{\mathrm{clean}}\left(t\right)\right\rangle }_{t=10..15\min}$. Insets: Snapshots of the beads in the entire 9×9mm chamber at t=20 min.

First, the bead surface fraction $\Psi_{b}$ on the bottom surface is measured in a target region we aim to clean. This region is a rectangle of 6×0.9mm, corresponding to 67×10% of the entire chamber, located 50μm away from the wall opposite to the light stimulus (see green rectangle in left inset of Figure 4a). Normalizing $\Psi_{b}$ by its initial value $\Psi_{b}^{0}$ at t=0, right before the blue light is switched on, ${\tilde{\Psi }}_{b}=\Psi_{b}/\Psi_{b}^{0}$ decreases consistently for all experiments, indicating that beads are being swept away from the target region, see Figure 4a. The decrease is very small for the smallest initial optical density ${\mathrm{OD}}_{i}=1$ ($c_{i}=3\times 10^{6}\;{\mathrm{cells}\;\mathrm{mL}}^{-1}$) with only 8% of the beads being swept in 20 min, whereas nearly all beads are moved away in this region of $\approx \;\;\;\;5\;{\mathrm{mm}}^{2}$ for an initial optical density ${\mathrm{OD}}_{i}=20$ ($c_{i}=6\times 10^{7}\;{\mathrm{cells}\;\mathrm{mL}}^{-1}$). The dynamics of cleaning likewise depend on the initial optical density. To estimate the characteristic time needed to clean the zone, the normalized fraction of beads ${\tilde{\Psi }}_{b}\left(t\right)$ is fitted to an exponential decay. The characteristic cleaning time τ decreases monotonically when the initial algal concentration increases, spanning from a couple of minutes at the highest concentrations to more than 300 min at ${\mathrm{OD}}_{i}=1$, see right inset in Figure 4a.

The bead surface fraction in a predefined region does not provide the full picture, as it does not account for the total impact of the bioconvective rolls. Thus, we introduce other metrics that take into account the whole extent of the bioconvection rolls effect on the passive beads. When a roll is formed, not all dense beads are pushed away in the experiments due to impeding phenomena such as surface friction on the channel floor, local stickiness, or variability in bead buoyancies. We define the cleaning efficiency ε to quantify the efficiency of the bioconvection roll for cleaning by comparing, in the entire region where the algae accumulated, the number of beads before and after the passage of the roll. Notably, the difference in beads is measured between t=0 and after the roll has completely vanished, to both avoid false negatives (some beads can be hidden by the high algal density) and also take the roll dispersion into account. The efficiency ε increases significantly with the initial seeding concentration, going from roughly 50%to80% when the initial OD goes from ${\mathrm{OD}}_{i}=1$ to ${\mathrm{OD}}_{i}=20$, see Figure 4b. This difference can be explained by the changes occurring in the rolls when the algal concentration increases: the rolls become both larger and faster when the initial OD rises (see Figure S11). In particular, these lower flow velocities could be too small to induce a motion of all beads, due to their interactions with the chamber floor. Note also that, while beads in a batch exhibit a range of buoyancies, between ≈1.001 and ≈1.009 with a median density ≈1.006, they are always denser than the initial solution of algae and sediment to the bottom of the chamber at the beginning of the experiment. They are then mostly swept away by the forming bioconvection roll before the local algal concentration reaches its maximum value. Beads remaining in the zone of the convection rolls could be those with a density smaller than the density of the local, highly concentrated algal region, or those that exhibit the most friction with the chamber floor.

As a last metric, the full cleaned surface $S_{\mathrm{clean}}$ is measured across the entire chamber. A surface is considered to be clean if there are strictly less than 2 beads in a 200×200μm region. Subtracting the initial value $S_{\mathrm{clean}}^{0}=S_{\mathrm{clean}}\left(t=0\right)$ before the blue light is switched on, we obtain ${\tilde{S}}_{\mathrm{clean}}\left(t\right)$ the curve shown in Figure 4c. The total surface cleaned by the algae ${\tilde{S}}_{\mathrm{clean}}^{\mathrm{tot}}$ is defined as the average value between 10 and 15 min. As for previous metrics again, the total surface cleaned increases monotonically with the initial algal concentration, ranging from $0.8\;{\mathrm{mm}}^{2}$ at ${\mathrm{OD}}_{i}=1$ to nearly $9\;{\mathrm{mm}}^{2}$ at ${\mathrm{OD}}_{i}=20$, i.e. more than 10 % of the chamber, see Figure 4d. This difference can be explained by the fact that, at lower concentrations c, algae are not sufficiently numerous to form a thick dense region. As a consequence, the convection roll remains at the wall. At larger concentrations, the dense region grows until all algae have accumulated and the convection roll moves away from the wall, following the algae front (see Movie S4). The total zone swept by the convection roll is thus the width of the roll itself plus the width of the dense algae region.

**FIGURE 5:**
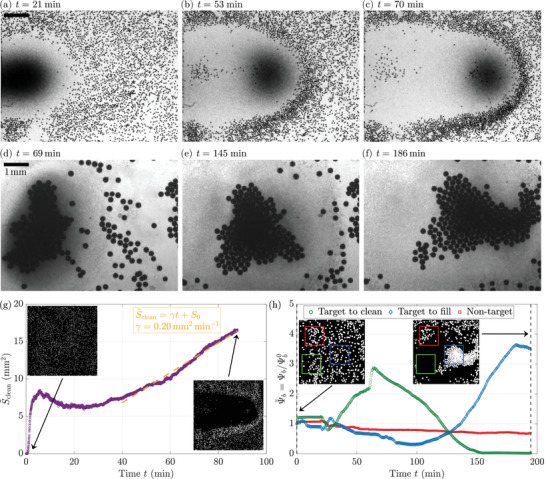
Large‐scale transport of large particles. (a–c) An “algae cannonball” cleans the chamber. Time‐lapse of an experiment with 50μm beads in a dense *C. reinhardtii* suspension at ${\mathrm{OD}}_{i}=7$ (top view). Both lateral LEDs are switched on, creating a concentrated algal plume far from the walls. Denser beads are pushed away from the plume as it moves forward, see Movie S5. In less than an hour, a region stretching over 4×5mm is almost perfectly cleaned from beads. (d–f) Transporting a raft of beads. Time‐lapse of an experiment with 230μm beads in a dense *C. reinhardtii* suspension at ${\mathrm{OD}}_{i}=10$ (top view). By carefully adjusting LED intensities, the algal plume and bead raft are gradually moved, displacing the raft by almost 4 mm over two hours. The latter collects neighboring beads as it moves, see Movie S6. (g) Temporal evolution of the cleaned surface ${\tilde{S}}_{\mathrm{clean}}\left(t\right)=S_{\mathrm{clean}}\left(t\right)-S_{\mathrm{clean}}^{0}$ by the “algae cannonball” shown in (a–c). Orange dashed line: affine fit ${\tilde{S}}_{\mathrm{clean}}=\gamma t+S_{0}$ with $\gamma =0.2\;\;{\mathrm{mm}}^{2}\;\min^{-1}$ and $S_{0}=-1.2\;\;{\mathrm{mm}}^{2}$. (h) Temporal evolution of the bead surface fraction on the top surface $\Psi_{b}$ for the beads raft shown in (d–f), normalized by its initial value $\Psi_{b}^{0}=\Psi_{b}\left(t=0\right)$ before the blue light is switched on. ${\tilde{\Psi }}_{b}$ is measured in three different regions of 7 mm^2^: a target region to clean (green), a target region to transport the beads to (blue) and a control region (red) as reference. Their respective locations are shown in insets.

To further bring the surface cleaning potential out, we place 50μm beads in a dense *C. reinhardtii* suspension at ${\mathrm{OD}}_{i}=7$ with both lateral LEDs on, forming a concentrated algal region away from the chamber walls. Axisymmetric convection rolls develop within a region of ∼1 mm radius, pushing beads outward within a larger influence zone of ≈2 mm, see Figure 5a and Movie S5. By gradually adjusting the intensities of the LED bands, the algal plume is moved across the chamber, see Figure 5b. As the plume advances, it pushes denser beads away, effectively cleaning the chamber floor. Within an hour, an area of approximately 4×5mm is nearly free of beads, see Figure 5c. The cleaned surface $S_{\mathrm{clean}}$ is further quantified as previously. Two regimes arise from its temporal evolution, see Figure 5g: at first, the cleaned surface quickly increases as the algal plume appears; then, it expands at a much slower rate, but consistently, as the bioconvection roll is directed toward the right side of the chamber. The cleaning rate observed on this curve is $\gamma =0.20\;\;{\mathrm{mm}}^{2}\;\min^{-1}$, a direct consequence of the approximately constant speed of the “algae cannonball” ($v_{c}\approx 0.1\;\mathrm{mm}\;\min^{-1}$), and the approximately constant size of the exclusion zone ($d_{c}\approx 2\;\mathrm{mm}$), yielding a rate $v_{c}\cdot d_{c}\approx 0.2\;\;{\mathrm{mm}}^{2}\;\min^{-1}$ identical to the measured rate. By the end of the experiment, after ≈80 min, more than 16 mm^2^ are completely cleaned of beads. This performance is quite impressive, considering the size of the microorganisms involved is around 10μm.

Bead behavior in response to light‐induced bioconvective flows depends on their relative density with the medium: lighter beads are attracted to high‐concentration regions, while denser beads are pushed away. To illustrate the potential for directional transport in targeted delivery, buoyant 230μm beads are placed in a dense *C. reinhardtii* suspension at an initial optical density ${\mathrm{OD}}_{i}$ of 10. Both lateral LEDs are switched on with different intensities, creating a highly concentrated algae plume near the left wall, see Figure 5d and Movie S6. This plume exerts a strong attraction on floating beads, capturing them as the convection rolls develop and eventually forming a large aggregate, as can be seen in Figure S14. By gradually adjusting the LED intensities, with incremental steps spread over two hours, we move the algal plume across the chamber, pulling the raft of beads along its path, see Figure 5d–f. The raft grows by collecting beads along its path, reaching the opposite side of the chamber after two hours, having covered a distance of approximately 4 mm and nearly doubling in size.

To quantify the system performance, we measure the bead surface fraction $\Psi_{b}$. Three different regions of area 7 mm^2^ are defined: a target region along the transport path, where beads should only travel transiently, a target region at the end of the transport path, where beads should end up accumulating, and a control region away from the transport path, which should not show any bead transport. These regions are shown in green, blue and red, respectively, in the inset in Figure 5h. The normalized bead surface fractions ${\tilde{\Psi }}_{b}$ are shown in Figure 5h for the three regions. After a transient step where beads are temporarily gathered in the region to clean as the raft is formed, see Figure 5d, the raft is directed to its target, the blue region. As such, the normalized bead surface fraction in the region to clean (green) falls to 0 while it consistently increases to reach more than three in the target region to fill (blue). The number of beads in the control region (red) stays relatively put, only exhibiting a slight decrease as a few beads are attracted by the algal bioconvection roll. The consistent growth of the raft highlights the system's ability to gather and transport passive objects within the active suspension, allowing us to direct the algal plume, and thus the bead raft, by precisely controlling LED intensities.

These observations and performance metrics demonstrate the potential for using algae‐induced flows to manipulate passive objects in biological suspensions, paving the way for future research and industrial applications, such as micropollutant cleaning, demixing of impurities (see Movies S7 and S8 for simulations highlighting the demixing potential of the system) and particle sorting in microfluidic devices (see Movie S9).

## 3 Discussion

To summarize, by stimulating a suspension of *Chlamydomonas reinhardtii* with light, we are able to transport hundreds of large passive particles over millimeters in a controlled manner. Transport is due to the formation of bioconvection rolls as algae, which are denser than water, accumulate horizontally in response to the light stimulus. Passive beads are either attracted or repelled by the rolls depending on their density with respect to the fluid, see Figure 1. The collective transport of beads is here mediated by the convective fluid flow, and not by steric interactions between individual algae and beads as in previous reports [9, 14, 15, 16]. We show that a minimal numerical continuous model of phototactic bioconvection quantitatively captures most of the experimental observations, see Figures 2 and 3. The bioconvection rolls are located at the edges of the concentrated algal region and extend over the area where there is a gradient in cell concentration, see Figure 2b. This concentrated region grows over time following simple scalings (see Figure 3d), and leads to up to 80 % of dense beads being swept away from the zone of bioconvection, with a clean zone of up to 8 ${\mathrm{mm}}^{2}$, see Figure 4. The zone concentrated in algae can be dynamically moved by tuning the light stimulus, which leads to beads being swept away or accumulated along the path of the moving blob of algae (see Figure 5 and Movies S5 and S6). The zone of attraction or repulsion of the beads is determined by the roll size, a few hundred micrometers with our experimental parameters. Three different quantitative metrics show that, the larger the algae concentration, the larger the surface cleaned, and the faster and more efficient the cleaning (Figure 4).

The setup described here builds upon previous photo‐bioconvection experiments [25, 27, 28] to drastically improve its applicability for cargo transport. Creating a lateral gradient of cell concentration as in ref. [25] instead of a vertical one suppresses the cell concentration threshold required in ref. [27] to trigger bioconvection. The use of phototaxis in the blue spectrum of light enables us to significantly reduce the time needed to establish bioconvection from approximately an hour in ref. [25] to about a minute, as in ref. [27]. Note that the algae accumulation observed in our experiments stems from negative phototaxis. Positive phototaxis would lead to accumulation at the wall close to the light stimulus, and should also trigger the formation of bioconvection rolls. However, in this positive phototaxis configuration, at high cell concentrations, microalgae close to the wall may screen the light stimulus. Algae in the chamber would then be prevented from seeing the phototactic light, which would limit the size of the accumulated region, which is itself directly linked to the strength of the bioconvection rolls. We therefore expect collective transport to be more efficient when using negative phototaxis.

Harnessing macroscale bioconvective flows for microscale cargo transport is an appealing path. These flows enable the simultaneous displacement of hundreds of particles, each of which can be an order of magnitude larger than a single microorganism, and can either attract or repel particles depending on their relative density with the fluid. Besides, this directional transport can be dynamically tuned with external stimuli, e.g. light, controlling the microorganism ‘taxis’. These properties, absent from single‐cell transport and multi‐cell surface patterning methods, pave the way for more complex control strategies of microscale transport by microorganisms. Here, we demonstrate how to control the bioconvective flows of *C. reinhardtii* suspensions with light, but the method is likely generalizable to other microorganisms (e.g. bacteria and other algae) and other external stimuli (e.g., chemicals [39] and magnetic fields [40]). As long as the stimulus produces a lateral accumulation of microorganisms, similar sustained bioconvective flows will emerge.

Finally, in addition to cargo transport, these bioconvective flows can be used for both mixing and demixing. On the one hand, for colloidal particles or molecules, bioconvection enhances mixing. In Nature, this improves the distribution of nutrients and oxygen as well as light penetration [41, 42, 43, 44]. Controlling these bioconvective flows in biotechnological applications such as bioreactors could reduce operational costs and preserve cell viability by reducing mechanical steering and the associated shear stress felt by the cells [27, 45, 46]. On the other hand, bioconvection can be used to separate non‐Brownian particles of different sizes and densities. By attracting light beads and repelling dense ones with a size dependent interaction strength, bioconvection rolls have the potential to demix heterogeneous suspensions of particles.

## 4 Experimental Section

### 4.1 Solution Preparation

*Chlamydomonas reinhardtii* strain CC‐125 (obtained from the Chlamydomonas Resource Center, University of Minnesota, MN, USA) was maintained by culturing on a solid medium consisting of Tris‐Acetate‐Phosphate (TAP, Gibco from ThermoFischer Scientific, France) and 1.5 % agar, with regular subculturing every 4 weeks. This cultivation method ensured the strain's motility, responsiveness to light, and minimized cell adhesion to surfaces. Liquid cultures were prepared every few days by inoculating algae from the solid culture into liquid TAP medium. These cultures were then incubated at 176 rpm, under a 14‐h light/10‐h dark cycle, with a light intensity of $60\;\mu \mathrm{mol}\;m^{-2}\;s^{-1}$, and maintained at a constant temperature of 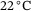. Maximum cell motility was typically reached within 3 days [38], resulting in a suspension of actively swimming *C. reinhardtii* cells at around $40\;\mu \;m\;s^{-1}\;\;\mathrm{to}\;\;60\;\mu \;m\;s^{-1}$ and a diffusion coefficient $D≃4\pm 1\times 10^{-9}\;\;m^{2}\;s^{-1}$ consistent with the literature [25, 26, 27, 29, 47, 48] (see Section SI.C.).

Prior to experimental use, the liquid cultures were centrifuged to concentrate the algal suspensions, eliminate low‐motility and dead cells, and remove cellular debris. In the first step, 45mL of the liquid culture was centrifuged at 1057 g for 10 min. In the second, 39mL of the supernatant was discarded, and the remaining 6mL was centrifuged at 73 g for 2 min. The resulting supernatant, containing the motile cells, was centrifuged once more at 285 g for 5 min and the required amount of supernatant was removed to keep a solution at a specific concentration. Then, a TAP solution containing blue polyethylene microspheres (Cospheric, US), with diameters $d_{b}$ ranging from 50μm to 460μm, was added to the suspension of algae. The particle concentration was tuned to ensure a good repartition of the beads for imaging. Surfactant (Tween 20, Sigma–Aldrich) was added to the solution at a concentration of 0.5 % to render the beads hydrophilic. We measured the densities of 50μm beads by placing them in solutions of salt of different concentrations. The beads were found to have a range of densities, between ≈1.001 and ≈1.009, with a median density ≈1.006.

### 4.2 Experimental Setup

Experiments were conducted within square chambers of width 9mm and height H=310μm (Frame–Seal in situ PCR and Hybridization Slide Chambers, Bio‐Rad), placed on top of a glass slide which was spin‐coated with polydimethylsiloxane (PDMS, Dow‐Corning Sylgard 184). The PDMS was made hydrophilic by plasma cleaning. Then, 32μL of the algal solution, mixed with beads, was deposited within. The chamber was gently closed with a plasma‐activated square PDMS lid. Experimental images were taken with a binocular (Leica MZ 16 FA) with a 1.0× objective (Plan APO) at 0.5fps. Since the algae do not react to red light, a red LED panel with wavelength λ=630nm (TX Series Backlight, Metaphase lighting technologies) and a monochrome camera (IDS U3‐3080CP‐M‐GL) were used for imaging. Two blue LED strips (Optonica, λ=470nm) of length 7.5cm were placed 3cm away from the assay, one on each side. The light intensity was tuned by varying the applied voltage and measured using a digital light sensor (Adafruit TSL2591). The resulting gradient in the y direction was measured for different distances between the LED band and the chamber, see Figure S2. The experimental setup is sketched in Figure 1a,b. Initially, the blue‐light is turned off and algae swim in all directions, populating the chamber homogeneously. In the experimental images, algae appear as a gray background, while the beads show up as large black dots, as shown in Figure 1d,h and Figure S1b. For large particles, the chamber height H was increased with more chamber seals such that $d_{b}<H/2$ to allow free movement of beads of different sizes. To achieve an accurate measurement of algal concentrations, we correlated optical density (OD) measurements, which we use as a proxy, with the grayscale values obtained from 8‐bit images, see Figure S3.

### 4.3 Numerical Simulations

Numerical simulations of the continuum model, Equations (1)–(3), are run in COMSOL Multiphysics v6.2. The numerical domain consists of a (y,z) slice of the experimental chamber of length 9mm and height 310μm. The Boussinesq Navier‐Stokes equation was handled with the laminar flow module with P2+P1 elements and a volume force, while the active advection diffusion equation was handled with the general form PDE module with quadratic Lagrange elements. A triangular mesh with quadratic elements for the boundary layers was used. Mesh element sizes range between 5μm and 18μm. At the chamber walls, we enforce the no‐slip boundary condition for the fluid velocity and no‐flux for the algae concentration c. In practice, we simulate the rescaled algae concentration $\bar{c}=c/c_{\max}$, which varies between 0 and 1, such that Equation (3) reads

$$\frac{\partial \bar{c}}{\partial t}+\nabla \cdot \left[\left(u-u_{\mathrm{photo}}\left(1-\bar{c}\right)e_{y}\right)\bar{c}\right]=D\Delta \bar{c}.$$

The density dependence in Equation (1) with this rescaled concentration was then $\rho \left(\bar{c}\right)=\rho_{w}+\bar{c}\left(\rho_{\max}-\rho_{w}\right)$ with $\rho_{\max}=\rho \left(c=c_{\max}\right)$. Equations are solved with the default fully coupled direct solver (MUMPS) and a relative tolerance of $10^{-3}$.

When comparing experiments and numerical simulation, we make sure to initiate the computation with the algae concentration profile observed experimentally just before the light was turned on. In experiments, we only have access to the algae concentration averaged over the chamber thickness $c\left(x,y,t\right)={\left\langle c\left(x,y,z,t\right)\right\rangle }_{z}$. We thus use this average value as the initial condition for the concentration at every z: c(y,z,0)=⟨c(y,0)⟩. We observe in experiments that the maximum concentration, and thus maximum density $\rho_{\max}$ increases slightly with the initial algae concentration in the range $1006\;\mathrm{kg}\;m^{-3}<\rho_{\max}<1009\;\mathrm{kg}\;m^{-3}$. The minimal optical density also does not always reach zero, indicating that some algae do not respond to light. We account for these non‐motile algae by adjusting $\rho_{w}$ in the range $1000\;\mathrm{kg}\;m^{-3}<\rho_{w}<1000.7\;\mathrm{kg}\;m^{-3}$. As a first approximation, the value of $u_{\mathrm{photo}}=35\;\mu \;m\;s^{-1}$ provides a good adjustment of the model for all initial algae concentrations.

To simulate the bead trajectories inside the photo‐bioconvective flow, we assume the dense beads of diameter $d_{b}=50\;\mu \;m$ to be passive tracers settled at the bottom of the chamber (z=0). At each numerical time step dt=0.5s, each bead j was advected at a velocity equal to the average horizontal fluid velocity over the bead diameter. Starting from an initial position $y_{b}^{j}\left(t=0\right)=y_{i}^{j}$ measured in experiments (see Figure 2a), we thus have $y_{b}^{j}\left(t+dt\right)=y_{b}^{j}\left(t\right)+v^{j}\left(t\right)dt$ with $v^{j}\left(t\right)=\left(1/d_{b}\right)\int_{0}^{d_{b}}u\left(y_{b}^{j}\left(t\right),z,t\right)\cdot e_{y}dz$.

The front speed was calculated from the position of the roll at 80% of its maximum position reached in 20 min: $v_{\mathrm{front}}^{\mathrm{sim}}=\frac{0.8\;y_{\max}-y_{i}}{t(y\;=\;0.8\;y_{\max})}$ This enables to take into account only the part of the data where the front speed was constant.

In Movie S4, we show the simulation reproducing the experiment with ${\mathrm{OD}}_{i}=10$, including a simulated dense bead of $d_{b}=50\;\mu \;m$. Movie S7 shows the simulation reproducing the experiment with ${\mathrm{OD}}_{i}=20$, including several dense (black) and buoyant (white) beads of $d_{b}=50\;\mu \;m$ randomly placed in the chamber to highlight the density based separation application. In Movie S8, we numerically reproduce the cannonball experiment by adjusting $u_{\mathrm{photo}}$ as a function of time, to mimic the adjustment in light intensity from the two light bands in the experiment. Movie S9 is a 3D generalization of Movie S8 illustrating particle transport in complex geometries. To simulate friction, a velocity threshold $u_{\mathrm{tresh}}=0.5\;\mu \;m\;s^{-1}$ was included for the bead movement in Movies S7–S9. For more details, see Supporting Information.

## Author Contributions

J.B. and M.J. conceptualized the research. T.L, J.B., M.J. and G.A. designed the experiments. T.L. and J.B. performed the experiments and analyzed the data. E.J.‐P. conceived and performed the numerical simulations. All authors discussed experimental and numerical results. G.A. and M.J. secured funding. T.L., J.B. and G.A. wrote the original draft of the manuscript. All authors reviewed the manuscript.

## Conflicts of Interest

The authors declare no conflicts of interest.

## Supporting information


**Supporting File 1**: smll72589‐sup‐0001‐SuppMat.pdf.

**Supporting File 2**: smll72589‐sup‐0002‐Movie1‐Repulsion.mp4.

**Supporting File 3**: smll72589‐sup‐0003‐Movie2‐Attraction.mp4.

**Supporting File 4**: smll72589‐sup‐0004‐Movie3‐Bouncing.

**Supporting File 5**: smll72589‐sup‐0005‐Movie4‐Simulation.mp4.

**Supporting File 6**: smll72589‐sup‐0006‐Movie5‐Raft‐transport.mp4.

**Supporting File 7**: smll72589‐sup‐0007‐Movie6‐Cleaning‐floor.mp4.

**Supporting File 8**: smll72589‐sup‐0008‐Movie7‐Sim‐Demixing.mp4.

**Supporting File 9**: smll72589‐sup‐0009‐Movie8‐Sim‐Cannonball.mp4.

**Supporting File 10**: smll72589‐sup‐0010‐Movie9‐Sim‐transport3D.mp4.

## Data Availability

Data are available from the authors upon reasonable request.
