# Supplementary material for: Controlling the Collective Transport of Large Passive Particles With Suspensions of Microorganisms
Source: Small. 2026 Feb 27;22(18):e11502. doi: 10.1002/smll.202511502 (PMC13014229; doi:10.1002/smll.202511502)
Supplement: Supplementary file 1 — Supporting File 1: smll72589‐sup‐0001‐SuppMat.pdf. [file SMLL-22-e11502-s009.pdf]

Supplementary Material – Controlling the Collective Transport of Large Passive Particles With Suspensions of Microorganisms

Taha Laroussi,[1,*] Julien Bouvard,[1,2,†] Etienne Jambon-Puillet,[1] Mojtaba Jarrahi,[3,‡] and Gabriel Amselem[1]

[1] *Laboratoire d'Hydrodynamique (LadHyX), CNRS, Ecole Polytechnique, Institut Polytechnique de Paris, 91120 Palaiseau, France*

[2] *Living Systems Institute, University of Exeter, Exeter, EX4 4QD, United Kingdom*

[3] *Université Paris-Saclay, CNRS, FAST, 91405 Orsay, France*

(Dated: January 30, 2026)

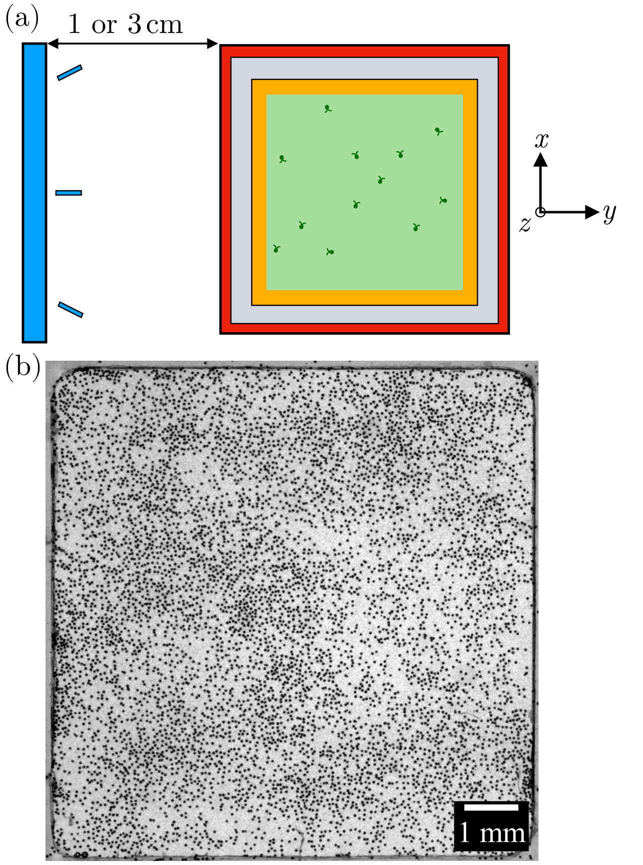

Figure S1. **Top view of the setup.** (a) Sketch of the $(9 \times 9)\,\mathrm{mm}^2$ chamber filled with a suspension of *Chlamydomonas reinhardtii* and passive polyethylene (PE) beads. The chamber is put on a PDMS-coated glass slide, on top of a red LED panel. (b) Top view of a chamber containing a suspension of *C. reinhardtii* at an optical density $\mathrm{OD}_i = 7$ (i.e., $c_i = 2.1 \times 10^7$ cells mL^{-1}) mixed with PE beads of diameter $d_b = 50\,\mu\mathrm{m}$.

SI. METHODS

A. Light intensity gradient within the chamber

Phototactic stimuli are provided by LED strips (LED SMD2835, Silamp). Each strip consists of five 2.8×3.5 mm blue LED ($\lambda = 470\,\mathrm{nm}$), equally spaced over 7.5 cm, see Figure S1. The local light intensity within the chamber is estimated with a two-step process. First, the light intensity emitted by the LED strips is measured directly on both sides of the chamber using a digital light sensor (Adafruit TSL2591). Then, to measure the light intensity perceived by algae within the chamber, the latter is filled with a solution of culture medium (Gibco™ TAP, ThermoFisher Scientific, France) supplemented with 5 % fluorescein. When the blue LEDs are turned on, fluorescein is re-emitting light with a peak at 520 nm, at an intensity proportional to the received blue light stimulus. The fluorescence signal is recorded with a fluorescence stereomicroscope (Leica MZ 16 FA) with a 1.0× objective (Plan APO) at 1900 ms exposure time. Pixel gray values are proportional to the emitted fluorescence, and are used as a proxy to measure the light intensity profile within the chamber. The absolute value of the light intensities within the chamber are then obtained by combining pixel gray values with the light intensities previously measured with the digital light sensor.

Different experimental setups were tested by varying the LED intensities and adjusting the LED strip's distance to the chamber, to achieve a light gradient predominantly in the y direction while minimizing the transverse gradient in the x direction. Our results indicate that both $\partial I/\partial x$ and $\partial I/\partial y$ across the chamber increases with higher LED intensities, see Figure S2 for the latter. Increasing the distance between the LED band and the chamber leads to a decrease in gradients, with a more pronounced reduction in $\partial I/\partial x$. Under identical experimental conditions, $\partial I/\partial x$ remains consistently lower than $\partial I/\partial y$: the gradient in the y-direction is higher even without an optimal setup. Based on these results, we find that the optimal configuration involves positioning the LED band 3 cm away from the chamber to minimize the light gradient in the x-direction. This setup maintains the longitudinal gradient, which is crucial for our experiments. In our experiments, we thus place the chamber 3 cm away from the LED band, with an applied voltage

* These authors contributed equally to this work.
† These authors contributed equally to this work.; Contact author: j.s.m.bouvard@exeter.ac.uk
‡ Contact author: mojtaba.jarrahi@universite-paris-saclay.fr

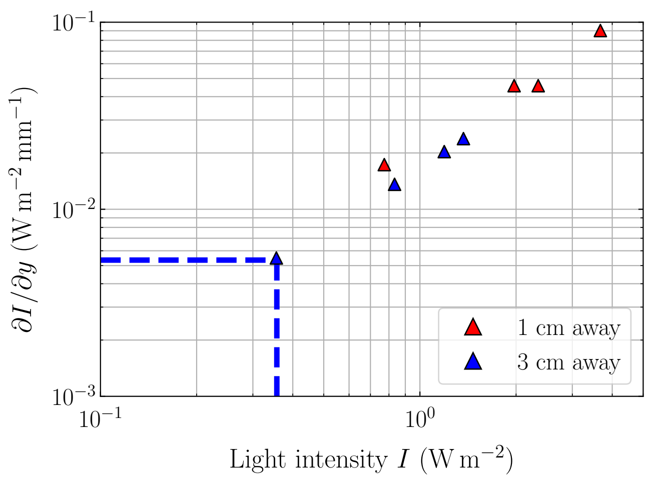

Figure S2. **Light intensity gradient within the chamber.** Light intensity gradient $\partial I/\partial y$ was measured for chambers positioned 1 cm (red markers) and 3 cm (blue markers) away from the blue LED band. Increasing the distance of the LED band to the chamber reduces the gradient. In our experiments, we place the chamber 3 cm away from the LED band. Under these conditions, we find an experimental light intensity $I \approx 0.35\,\mathrm{W\,m^{-2}}$, represented by the dashed blue lines.

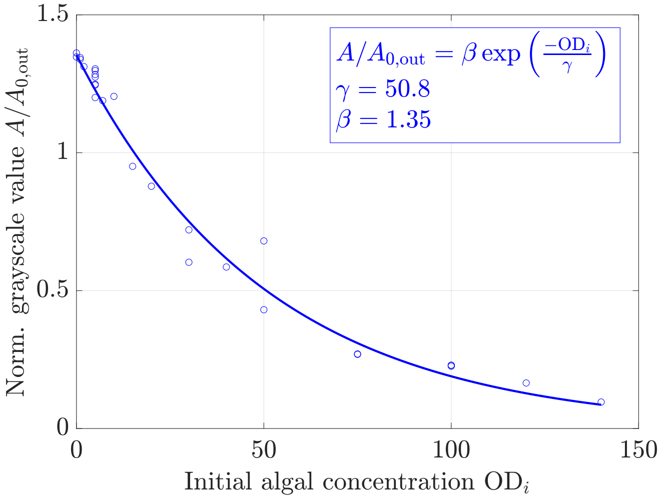

Figure S3. **Calibration of algal concentration measurements.** Normalized grayscale values inside the chamber are plotted against the corresponding initial algal concentrations measured in optical density (OD). For reference, $1\,\mathrm{OD} = 3 \times 10^6\,\mathrm{cells\,mL^{-1}}$. The concentration is measured with a spectrophotometer before filling the chamber with the algal solution. The blue line shows the best fit for the calibration.

of 10 V. Under these conditions, we find an experimental light intensity $I \approx 0.35\,\mathrm{W\,m^{-2}}$, represented by the dashed blue lines. These direct measurements allow for a more accurate assessment of the light intensity gradient compared to using gray values.

B. Calibration of algal concentration measurements

To achieve an accurate measurement of algal concentrations, we correlated optical density (OD) measurements with grayscale values obtained from 8-bit images. For reference, $1\,\mathrm{OD} = 3 \times 10^6\,\mathrm{cells\,mL^{-1}}$. We performed several calibration experiments, with varying algae concentrations, to map OD values to grayscale values. Before filling the chamber, the initial concentration of algae OD_i was measured using a spectrophotometer. After filling, we captured an image of a large region where algae are distributed homogeneously and calculated the mean gray value within the chamber A, and outside the chamber $A_{0,\mathrm{out}}$. To account for differences in experimental conditions, such as variations in red LED panel light intensity or camera exposure time, the gray values within the chamber were normalized by those outside. The resulting data was fitted with a decreasing exponential, similar to Beer-Lambert law, in the form:

$$A/A_{0,\mathrm{out}} = \beta \exp\left(-\frac{\mathrm{OD_i}}{\gamma}\right), \qquad (1)$$

with the best-fit parameters being $\gamma = 50.8$ and $\beta = 1.35$, see Figure S3. The calibration curve derived from these experiments provides a mapping between grayscale values and the local algal concentration in OD.

C. Algae motility

We characterize the motility of our algae *Chlamydomonas reinhardtii* CC-125 by measuring their swimming speed alongside their diffusivity. Their swimming speed is measured by tracking individual *C. reinhardtii* cells swimming in the bulk of a homogeneously filled closed chamber. Movies are recorded at 10 Hz for 30 s under a Nikon TI microscope in a dark room, with a red filter (Newport RG645) to avoid any phototactic effects, using a 10× objective and a CMOS camera (Hamamatsu ORCA-Flash4.0 LT, Hamamatsu Photonics, France). Tracking is performed in Fiji with the TrackMate plugin [1]. A typical distribution of the mean algal velocity along its trajectory is shown in Figure S4, highlighting a large variation of the algae swimming speed peaked around $u_{\mathrm{swim}} = 40 - 60\,\mathrm{\mu m\,s^{-1}}$.

To measure the diffusion coefficient of our algae *C. reinhardtii*, we concentrate them in a small region of the chamber utilizing their negative phototactic response to intense blue light. Once accumulated on one wall of the chamber, we switch off the blue light stimulus. The algae thus diffuse away from the dense region, the temporal evolution of the algal concentration profile being consistent with a diffusive behavior. By fitting an isoconcen-

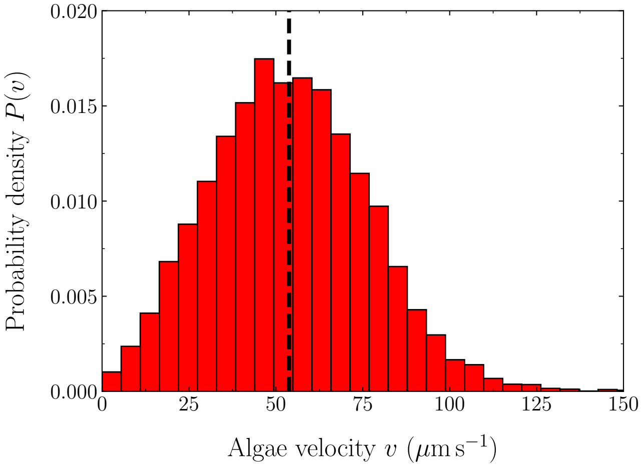

Figure S4. **Typical distribution of the mean trajectory velocity** of *C. reinhardtii* in 2D, for an experiment performed at OD = 1 and tracked for 30 s at 10 fps. The average swimming speed, displayed in dashed black line, is (54 ± 23) µm s^{-1}.

tration with

$$y(t) = y_0 + \frac{2\sqrt{D}}{\mathrm{erf}(1 - \mathrm{OD}_{\max}/\mathrm{OD}_i)}\sqrt{t}, \qquad (2)$$

we find the diffusion coefficient of the algae as $D = (4 \pm 1) \times 10^3\ \mu\mathrm{m}^2\,\mathrm{s}^{-1} = (4 \pm 1) \times 10^{-9}\ \mathrm{m}^2\,\mathrm{s}^{-1}$. This value is consistent with previous diffusivity measurements of *C. reinhardtii* which found $D = 8 \times 10^{-10} - 8 \times 10^{-8}\ \mathrm{m}^2\,\mathrm{s}^{-1}$ depending on the strain and culturing conditions [2–6], as well as the lighting conditions [7].

D. Bead front detection

As algae accumulate, they tend to push the beads closer together, forming a dense line, referred to as the 'front', see Figure S5a. Since the front may not be perfectly flat or parallel to the wall due to variations in the algae accumulation pattern, we divide the chamber into 13 vertical slices. This discretization of the front allows us to analyze each region independently. The bead distribution is then smoothed by applying a 20×20 uniform kernel to blur each slice. We then average the pixel intensity values along the x-direction within each blurred slice, producing a vertical profile of average gray values at each y-position. Since beads appear as dark spots in the images, the front is identified as the y-position where the pixel intensity reaches a local minimum, corresponding to the bead accumulation near the wall. These minimum positions represent the bead front for each slice and are shown as red vertices, see Figure S5b. The vertices from all slices are then connected with straight lines to reconstruct the continuous bead front. A locally weighted linear regression ("rlowess") is applied to smooth the front, shown in green, see Figure S5b. The vectors normal to this smoothed front, shown in blue, indicate the local direction in which beads are pushed. Front detection can be difficult close to the wall or at early times, especially at low initial algae concentrations. In such cases, algae accumulation is thin and the wall can sometimes be misidentified as the bead front. To address this issue, we define dynamic detection boundaries that evolve

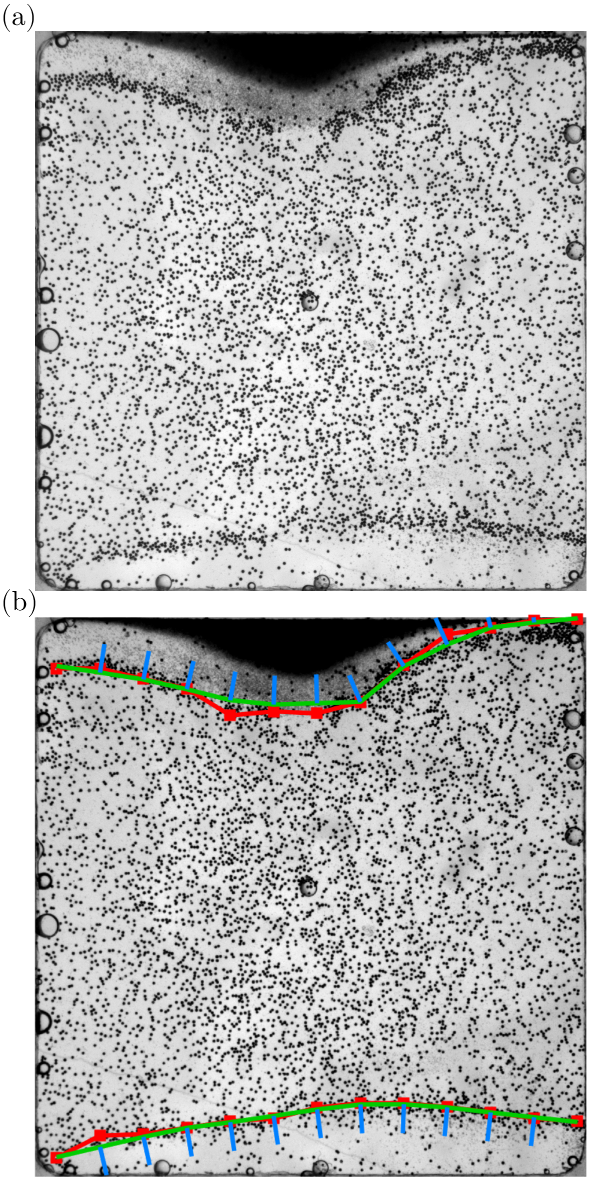

Figure S5. **Bead front detection**. (a) Image of the chamber with algae accumulated at the top boundary, pushing the beads away. Prior to this, the opposite LED was on, leading to the formation of another bead front near the bottom wall. The initial algae concentration is $\mathrm{OD}_i = 5$. (b) Vertices of the bead fronts for each vertical slice, connected by straight lines, are shown in red. The smoothed front is shown in green. Normal directions to the smoothed fronts, at each vertex, are shown in blue, indicating the local direction of bead displacement.

with time: the boundary at each time step is adjusted to the previous position of the bead front ± 20 pixels, improving the accuracy of front detection over time. Additionally, each experiment undergoes visual verification of the bead front detection. Algae tend to accumulate more uniformly in the chamber's middle section. Unreliable vertices, especially those near corners, are often removed. Indeed chamber corners refract light and interfere with algae accumulation. This process allows for a robust detection of bead fronts and study of their dynamics.

E. Numerical simulations - Supplementary Movies

In Movie S4, we show the simulation reproducing the experiment with $\mathrm{OD}_i = 10$ ($\rho_w = 1000\,\mathrm{kg\,m^{-3}}$, $\rho_{\max} = 1007.8\,\mathrm{kg\,m^{-3}}$), including a simulated dense bead of $d_b = 50\,\mu\mathrm{m}$. Movie S7 shows the simulation reproducing the experiment with $\mathrm{OD}_i = 20$ ($\rho_w = 1000.7\,\mathrm{kg\,m^{-3}}$, $\rho_{\max} = 1009\,\mathrm{kg\,m^{-3}}$), including several dense (black) and buoyant (white) beads of $d_b = 50\,\mu\mathrm{m}$ randomly placed in the chamber to highlight the density based separation application. In Movie S8, we numerically reproduce the cannonball experiment ($\rho_w = 1000\,\mathrm{kg\,m^{-3}}$, $\rho_{\max} = 1009\,\mathrm{kg\,m^{-3}}$, $\bar{c}_i = 0.15$). To do so, $\boldsymbol{u}_{\mathrm{photo}}$ is adjusted as a function of time, mimicking the adjustment in light intensity from the two light bands in the experiment. In practice, the algae concentration field is made to follow a target location $y_0(t)$ that is time dependent such that $\boldsymbol{u}_{\mathrm{photo}} = -u_{\mathrm{photo}}(1-\bar{c})\frac{(y-y_0)}{\sqrt{(y-y_0)^2}}\mathbf{e}_y$. In the movie, $y_0(t)$ is chosen as a piecewise function

$$y_0(t) = \begin{cases} 0 & \text{if } t \leq t_1, \\ v_c(t-t_1) & \text{if } t \geq t_1. \end{cases}$$

with $v_c = 2\,\mu\mathrm{m\,s^{-1}}$ and $t_1 = 200\,\mathrm{s}$. To simulate friction, a velocity threshold $u_{\mathrm{tresh}} = 0.5\,\mu\mathrm{m\,s^{-1}}$ was included for the bead movement in Movies S7, S8 and S9.

Movie S9 is a three dimensional generalization of Movie S8 illustrating particle transport in complex geometries. Equations (1)-(3) from main text are solved on a three dimensional geometry representing a microfluidic chip with three wells. The mesh is coarser (element size $\sim 50\,\mu\mathrm{m}$) and the parameters are $\rho_w = 1000\,\mathrm{kg\,m^{-3}}$, $\rho_{\max} = 1009\,\mathrm{kg\,m^{-3}}$, $\bar{c}_i = 0.5$ in one of the well and zero elsewhere. The simulated blob is moved in 3D with a 2D target point $\{x_0(t), y_0(t)\}$, such that $\boldsymbol{u}_{\mathrm{photo}} = -u_{\mathrm{photo}}(1-\bar{c})\frac{(x-x_0)\mathbf{e}_x+(y-y_0)\mathbf{e}_y}{\sqrt{(x-x_0)^2+(y-y_0)^2}}$. This target point is moved from one chamber to another with a velocity $v_c = 2\,\mu\mathrm{m\,s^{-1}}$. Such control of the algae could be achieved experimentally with a careful tuning of lights coming from six LED strips, two on each side of the three channels. Movie S9 shows a top view of the 3D simulation. The density is taken at the midplane, i.e. $\rho(z = H/2)$, and the velocities are taken at three quarters of the height, i.e. $\boldsymbol{u}(z = 3H/4)$. The motion of buoyant beads of diameter $d_b = 50\,\mu\mathrm{m}$ (white) randomly dispersed in two of the wells is simulated following the same procedure as in 2D, but for both the x and y components of the bead velocity.

SII. RESULTS

A. Trajectories of beads close to convection rolls

All experimental bead tracks are obtained with the TrackMate plugin [1] in Fiji. As highlighted in the sketch of Main Figure 1c, the bioconvection rolls created by algae accumulation cause the denser beads to be pushed away, while lighter beads are drawn into the convection rolls. The density of the beads determines whether they are repelled or drawn into the algae-induced flows.

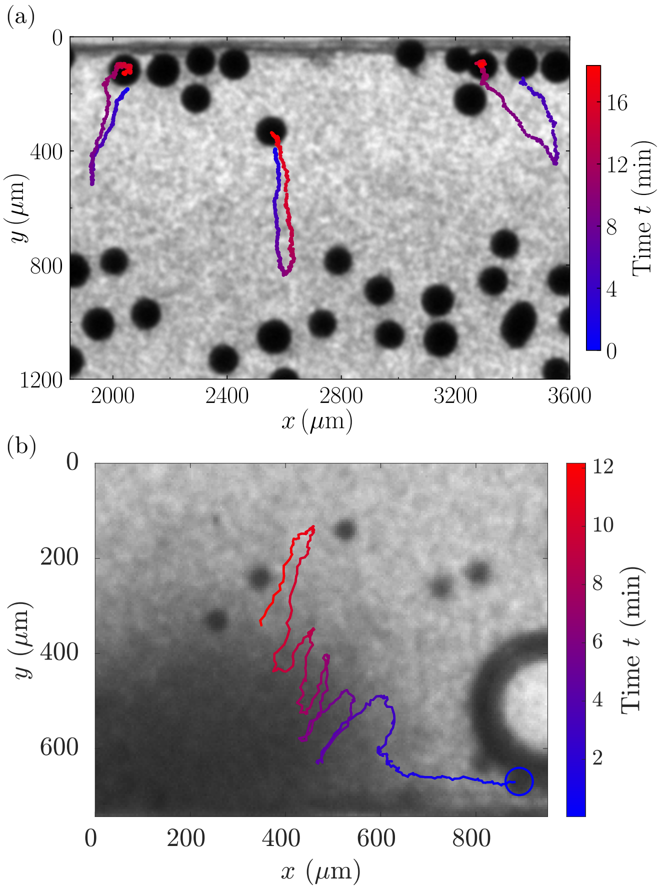

Figure S6. **Beads caught within convection rolls.** (a) Trajectories of three $115\,\mu\mathrm{m}$ diameter beads caught within convection rolls. Initially, the beads are pushed away from the algae accumulation region at the wall. After traveling a few hundred microns, they are drawn back toward the wall. (b) Trajectory of a $d_b = 50\,\mu\mathrm{m}$ bead trapped in a small convection roll. This bead exhibits multiple revolutions inside the roll, looking like it is 'bouncing' on the edge of the dense algal region for nearly 1 h, see Movie S3. The initial algal concentration for both experiments is $\mathrm{OD}_i = 10$.

Nevertheless, some beads closely match the density of the medium, allowing them to follow the flow patterns. At first, these beads are pushed away and move outward from the high algae density region, see Figure S6a. After traveling a few hundred micrometers, they enter upward-moving flows, which recirculate toward the wall, eventually drawing the beads back to the algae-dense region. This example highlights the simultaneous presence of inward (attractive) and outward (repulsive) flows at different heights within the chamber. As algae swim on average upward toward the wall, they displace the surrounding fluid, creating inward flows that transport beads. Once the algae accumulate, the unstable lateral concentration gradient causes the algae plume to sink, generating downwelling flows that push beads away from the high concentration zone. This cyclic movement is particularly visible for beads that closely match the density of the medium, as they experience minimal buoyancy, allowing them to follow the convection rolls and perform looping trajectories, see Figure S6b. Looking at this phenomenon from the top, it seems like the beads are 'bouncing' on the edge of the dense algal region, see Movie S3. For comparison between experimental and numerical results (Fig. 2d in the main text), all bead tracks starting at $t = 0$ were considered, for beads that never entered upwelling flows.

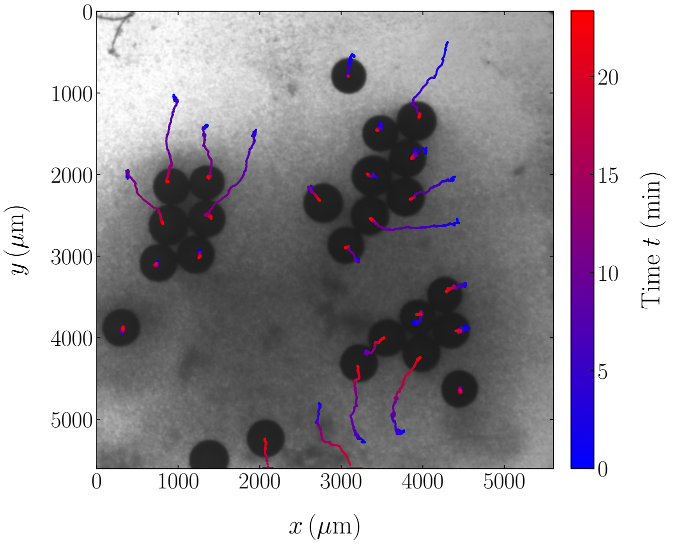

Figure S7. **Trajectories of large beads attracted by plumes of algae.** Multiple algal plumes appear, locally attracting large floating beads of diameter $d_b = 460\,\mu m$ in their vicinity. Snapshots of the left aggregate constituted of 6 beads, are shown in Main Figure 1f-k. See also Movie S2.

Using lighter beads of diameter $d_b = 460\,\mu m$, we can show different bead dynamics in the vicinity of algal plumes. When a large algal plume appears, it attracts the floating beads nearby (see Main Figure 1f-k and Movie S2). Trajectories of such beads are displayed in Figure S7.

On a longer time scale ($\sim 1\,h$), some floating 460 μm beads can 'surf' over the plumes, hopping on and off as they develop and disappear, allowing them to cover much longer distances that can reach 6-9 mm over 80 min, see Figure S8a. By zooming in on one of the beads, we see how it accelerates and decelerates twice as it goes through different convection rolls, reaching velocities as high as

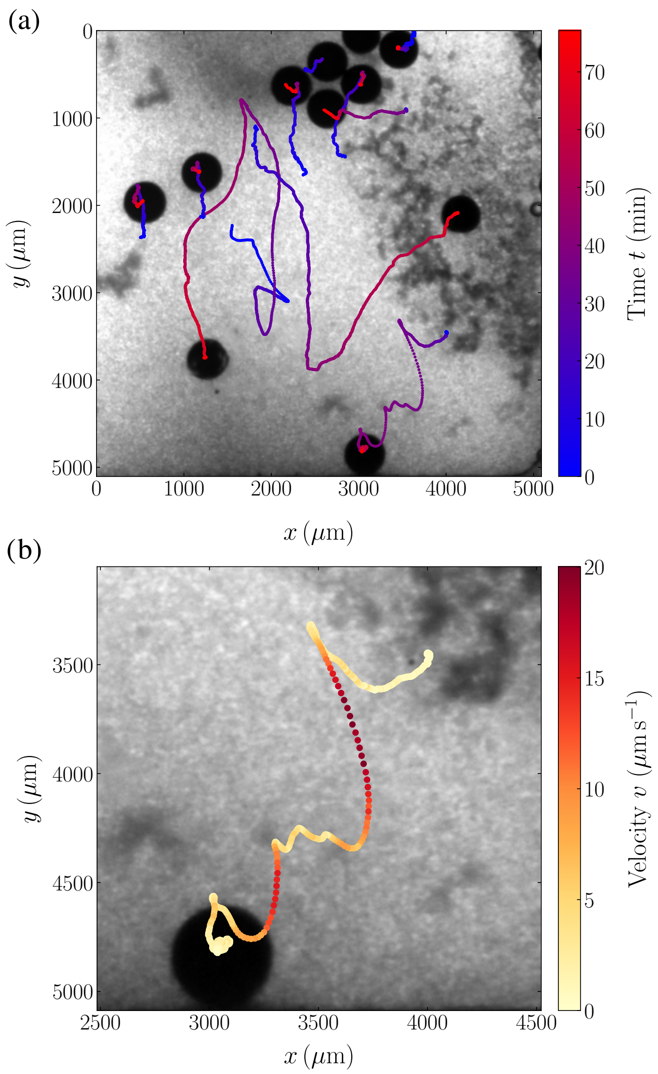

Figure S8. **Long trajectories of large floating beads caught in multiple algal plumes.** Complex algae patterns appear over the course of the experiment, locally attracting buoyant beads of 460 μm diameter in their vicinity. Depending on their location with respect to forming plumes, some beads can travel large distances (6-9 mm), hopping on and off as algal plumes develop and disappear. (a) Several beads of diameter $d_b = 460\,\mu m$ exhibit complex, long-range trajectories as they go through different convection rolls over 77 min. (b) A close-up view of a single large bead 'surfing' over forming algae plumes. This bead, of diameter $d_b = 460\,\mu m$, accelerates and decelerates twice as it goes through different convection rolls, reaching velocities as high as $20\,\mu m\,s^{-1}$ over 77 min. Trajectories in (a) and (b) are superimposed on the last image of the trajectory taken at $t = 77$ min.

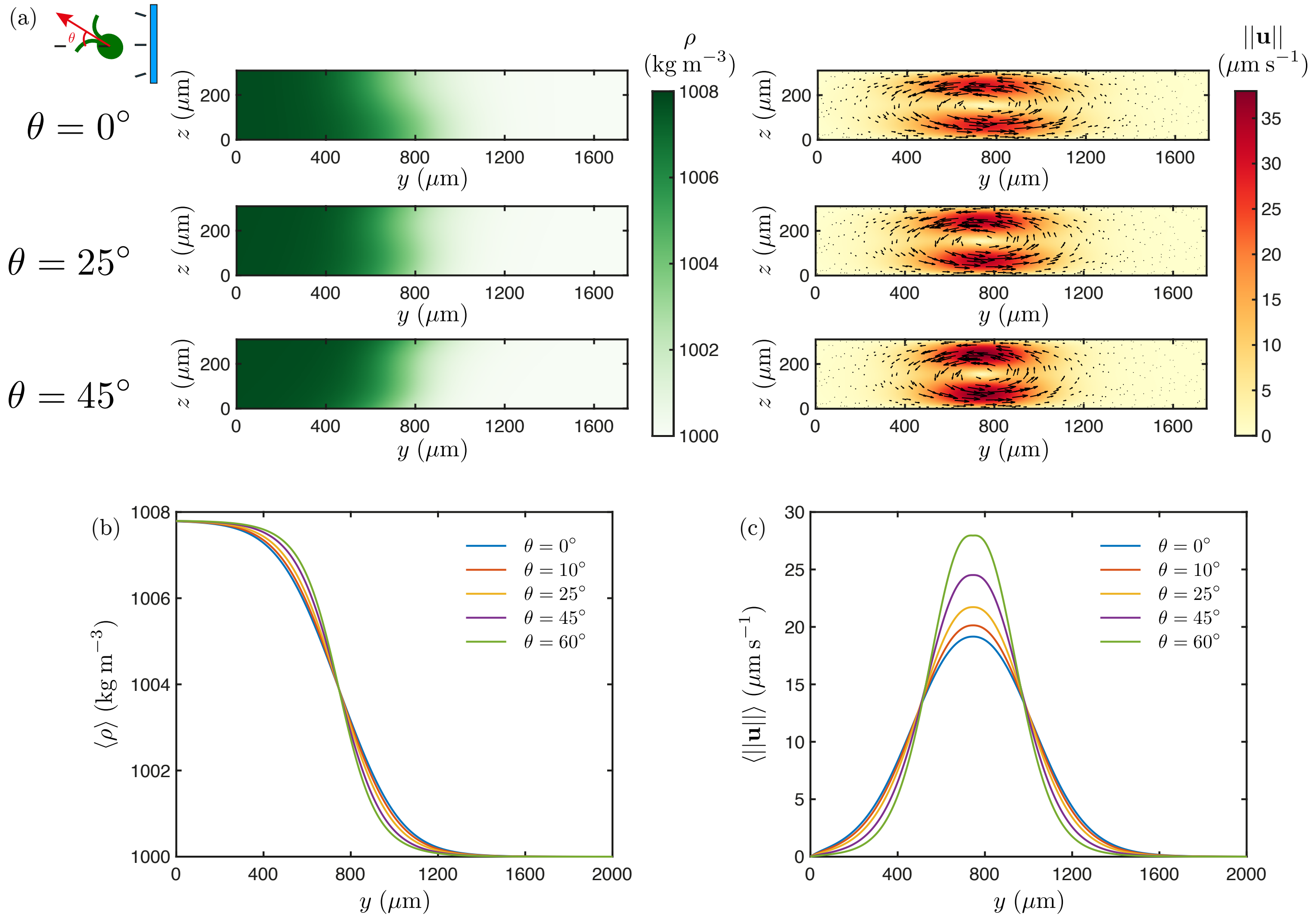

Figure S9. **Effect of gravitaxis in the continuum model.** (a) Density and velocity maps at $t = 480\,\mathrm{s}$ for simulations of the model with various values of the angle θ which introduces a gravitactic vertical velocity (see schematic on the top left and Eq. (3)). The horizontal velocity $u_{\mathrm{photo}} \cos\theta$ is kept constant for all simulations. (b,c) Depth averaged density $\langle\rho\rangle$ (b) and velocity magnitude $\langle||\mathbf{u}||\rangle$ (c) profiles at steady-state ($t = 800\,\mathrm{s}$) for different values of θ.

$20\,\mu\mathrm{m\,s^{-1}}$, see Figure S8b. This individual tracking approach highlights the complex response of beads to bioconvective flows, whether pushed away from algae-dense regions near the walls or drawn toward concentrated algal plumes.

B. Numerical simulations

In the minimal continuum model presented in Section II.B. of the main text, we neglected gravitaxis which is known to affect *C. reinhardtii* [8]. We can include it in the model by changing the orientation of the swimming velocity $\mathbf{u}_{\mathrm{photo}}$. Assuming that the algae swim on average upward with an angle θ with respect to the light direction $\mathbf{e_y}$, the phototactic velocity reads

$$\mathbf{u}_{\mathrm{photo}} = u_{\mathrm{photo}} \cos\theta \left(1 - \frac{c}{c_{\mathrm{max}}}\right)(-\mathbf{e_y} + \tan\theta\, \mathbf{e_z})\,. \quad (3)$$

We solve Equations (1)-(3) in main text using the phototactic velocity above in Equation (3) for various values of θ in the conditions of Figure 2 of the main text, while keeping the horizontal component of the velocity constant, $u_{\mathrm{photo}} \cos\theta = 35\,\mu\mathrm{m\,s^{-1}}$. The density and velocity maps for $\theta = \{0^\circ, 25^\circ, 45^\circ\}$ at $t = 480\,\mathrm{s}$ are shown in Figure S9a. A higher value of θ tilts the density front and increases the magnitude of convection flows. However, the effects are small, even for large values of θ for which gravitaxis dominates phototaxis. The steady-state depth averaged density and velocity profiles are shown in Figure S9b,c and confirms that the effect of θ is weak. Thus, gravitaxis is not critical to describe the phototactic bioconvection rolls observed in our experiment, and we discard it from the model presented in the main text.

The model introduces a crowding factor $(1 - c/c_{\mathrm{max}})$ in the swimming velocity $\mathbf{u}_{\mathrm{photo}}$ to restrict the algae concentration below c_{max}. While several physical mechanisms could explain a decrease in swimming velocity at high algae concentration (steric repulsion, local cell stresses,

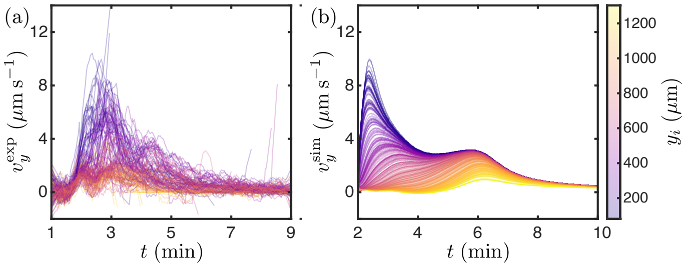

Figure S10. **Experimental and simulated bead velocity profiles.** Experimental (a) and numerical (b) velocities of all the beads shown in Figure 2d of main text as a function of time. The color codes the bead initial position y_i with respect to the chamber wall. $t = 0$ is defined as the time when the blue LEDs are switched on. Simulated profiles in (b) are shifted by $t_{\rm lag} \simeq 2\,\rm min$ to match the experimental profiles in (a), see main text.

light shielding, etc.), the choice of a linear dependence is arbitrary and was made for simplicity. Instead, the concentration dependency could be convex (i.e. strong at low concentrations and weak at high concentrations, like the light intensity in Beer-Lambert's law) or concave (i.e. weak at low concentrations and strong at high concentrations, like the viscosity of suspensions). We solved Equations (1)-(3) of the main text with a crowding factor of the form $(1 - c/c_{\rm max})^n$ to test the effect of both a convex ($n > 1$) and a concave ($n < 1$) phototactic velocity concentration dependency. In practice, for $n < 1$ the crowding factor was truncated at $c = 0.995\,c_{\rm max}$ to avoid numerical difficulties, i.e. $\lim_{c\to c_{\rm max}} ({\rm d}\mathbf{u}_{\rm photo}/{\rm d}c) = \infty$, and, for $n > 1$, $c_{\rm max}$ was slightly increased to reach the same density in the concentrated algal region. Qualitatively, the results are similar for all n: we always observe a density front and a convection roll. Quantitatively, the velocity magnitude decreases slightly with n and the front/roll is sharper for $n < 1$ and wider for $n > 1$. When comparing the simulated height averaged density profiles to experiments (e.g. Figure 3a of main text), varying n does not significantly improve the model, and therefore we keep $n = 1$ for simplicity.

A side-by-side comparison of the experimental and numerical temporal velocity profiles of all the beads in Figure 2d of main text is shown in Figure S10. The numerical profiles are in good agreement with the experimental ones, exhibiting a similar shape and order of magnitude. The beads initially close to the wall ($y_i \leq 500\,\mu$m, in purple) are initially advected away by the convection roll at speeds up to $\approx 10\,\mu\rm m\,min^{-1}$ for $\approx 1\,$min, before being slowly pushed away at $\approx 0.5\,\mu\rm m\,min^{-1}$. The beads initially far away from the wall ($y_i \geq 900\,\mu$m, in yellow) are displaced at velocities smaller than $\approx 4\,\mu\rm m\,min^{-1}$. Interestingly, even the two bumps that appear in the simulated profiles around $t \simeq 30\,$s and $t \simeq 4\,$min can be observed somewhat in the noisier experimental profiles.

The numerical simulations allow for a characterization of the size and intensity of the bioconvection rolls when the initial seeding concentration of algae varies. The width of the roll $L_{\rm roll}$ and its maximum velocity $u_{\rm max}$ are measured in the simulations, see Figure S11, for the four algal concentrations used in the experiments (OD$_i$ = 1, 5, 10 and 20). Both $L_{\rm roll}$ and $u_{\rm max}$ increase with the initial algal concentration, showing that the roll becomes both larger and stronger. Nevertheless, the variations are far from linear, with the roll size and the maximum velocity only increasing by respectively $\approx 35\,\%$ and $\approx 55\,\%$ when the optical density is increased 20-fold from OD$_i$ = 1 to OD$_i$ = 20.

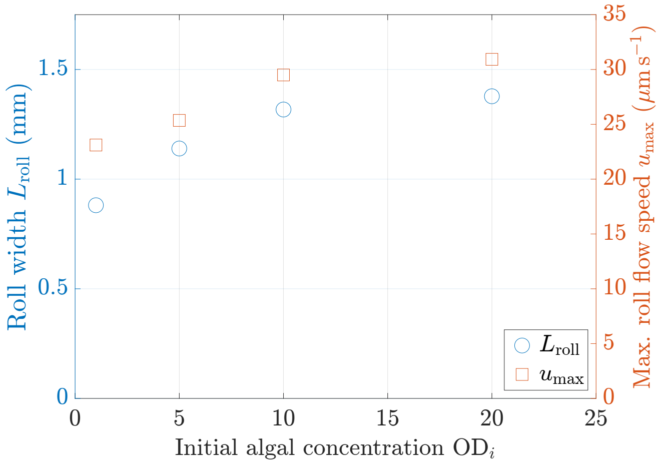

Figure S11. **Characteristics of simulated bioconvection rolls.** Width $L_{\rm roll}$ (blue) and maximum velocity $u_{\rm max}$ (orange) of the bioconvection roll, for different initial optical densities of algae, measured from the simulations. The roll width $L_{\rm roll}$ corresponds to the size of the roll in the y direction (perpendicular to the wall), using a fluid velocity of $0.5\,\mu\rm m\,s^{-1}$ as a threshold to define the roll edges.

C. Algae adaptation to light

Although the fast ballistic phase observed during $\approx 5\,$min can be readily attributed to the bioconvection rolls ejecting away the beads or attracting them, the existence of the last sustained slow regime in Figure 3c of main text, with a constant bead velocity $\approx 0.5\,\mu\rm m\,min^{-1}$ is more complex to explain. We turn to monitoring of algal accumulation in the absence of beads, to better visualize the different phases of the algal response.

When a lateral light source is turned on, the algae accumulate at the opposite wall and concentrate in a dense band. The algal concentration in the band increases from OD$_i$ = 10 to as high as OD $\approx$ 90. The size of the band also increases with time, reaching $\approx 500\,\mu$m after a dozen minutes of light stimulus, see Figure S12a. During the first dozen minutes, the band is very well defined, and we observe a sharp interface between the region dense in algae and the remaining of the chamber. After a dozen minutes, however, the initially sharp concentrated band both widens and blurs, an indication

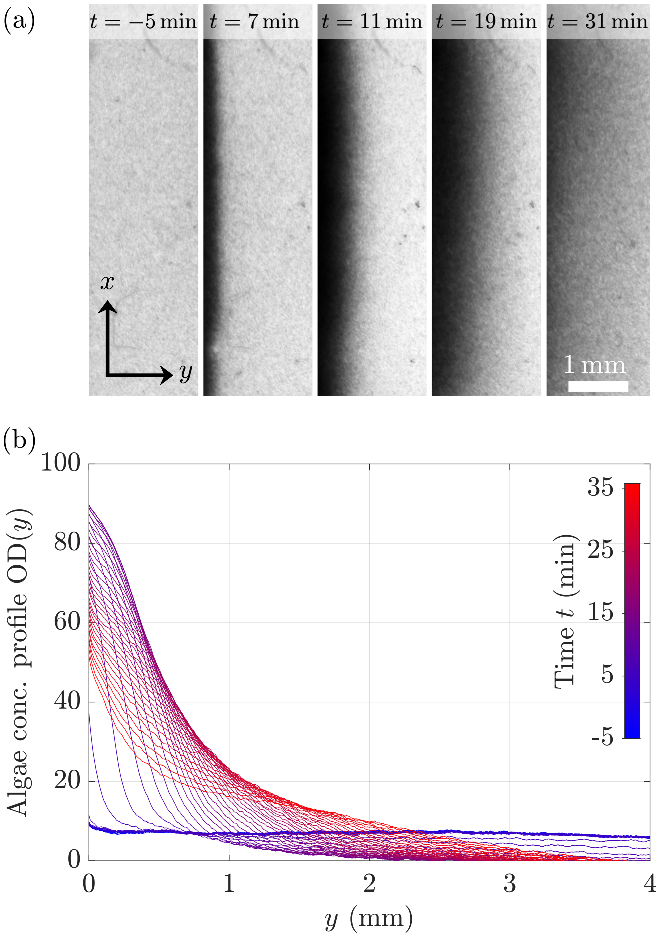

Figure S12. **Phototactic adaptation to sustained light exposition.** (a) Time-lapse of an experiment without beads where a *C. reinhardtii* suspension at $\mathrm{OD}_i = 10$ is exposed to a lateral light source coming from the right ($y > 9\,\mathrm{mm}$). Initially, algae are homogeneously distributed within the chamber. Once the lateral blue LED is switched on at $t = 0\,\mathrm{min}$, algae exhibit negative phototaxis and rapidly accumulate at the opposite wall ($y = 0$). By $t \approx 16\,\mathrm{min}$, a dense region of algae has formed, spanning several hundred microns. A stationary state is reached when phototaxis is balanced by diffusion. Then, the phototactic response weakens due to adaptation which causes the algae to diffuse back into the rest of the chamber. (b) Temporal evolution of the algal concentration profile near the wall. At $t \leq 0$, the profile is flat as algae are uniformly distributed. A high concentration region forms close to the wall. Over time, the profile flattens as the phototactic response weakens due to adaptation, while still maintaining a high concentration region close to the wall.

that algae start moving toward the light. After half an hour of light stimulus, the maximum algal concentration close to the wall is OD $\approx$ 50, but the band cannot be easily defined anymore. This blurring of the cell concentration can be attributed to cell adaptation to light, a well-known phenomenon [3, 9]. As cells adapt to light, their negative phototactic response dwindles, resulting in a decrease in their phototactic velocity u_{photo}. Thus, the algal concentration profile flattens with time, lowering the concentration gradient, see Figure S12b.

The adaptation of algae to light is therefore responsible for both the sustained bead motion and the slowing down of their velocity. Indeed, in the absence of adaptation, the beads would be ejected away from the bioconvection rolls and remain immobile at the boundary of the rolls, as can be seen in Movie S4 for simulations without adaptation. Adaptation leads to a widening of the zone of high algal concentration, and so of the zone of bioconvection, which pushes the beads further away from the chamber walls. At the same time, the gradient of cell concentration decreases, and so does the lateral density gradient responsible for bioconvection rolls. This reduction in the density gradient leads to a slowing of the flow speed in the bioconvection rolls.

The numerical simulations based on algae swimming

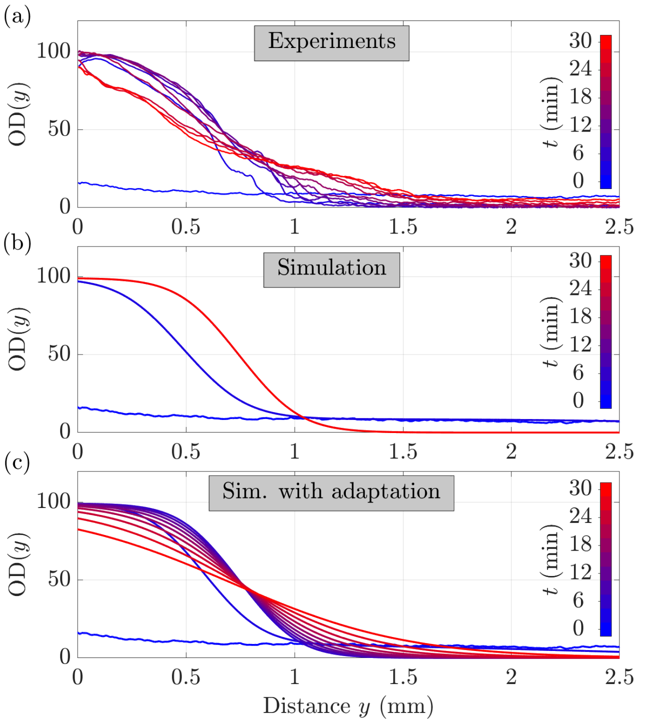

Figure S13. **Adaptation is needed in the numerical simulations to match the experimental algal concentration profiles.** (a) Temporal evolution of the experimental algal concentration profile $\mathrm{OD}(y)$ near the wall ($y = 0$) for an initial optical density $\mathrm{OD}_i = 10$. After the blue LED is switched on at $t = 0$ and $y > 9\,\mathrm{mm}$, the algae begin to accumulate at the wall. (b) Temporal evolution of $\mathrm{OD}(y)$ obtained from the numerical simulations with a constant $u_{\mathrm{photo}} = 35\,\mathrm{\mu m\,s^{-1}}$, i.e. without any light adaptation. (c) Temporal evolution of $\mathrm{OD}(y)$ obtained from the numerical simulations with light adaptation: $u_{\mathrm{photo}}(t) = u_{\mathrm{photo}}(0) - \alpha t$, with $u_{\mathrm{photo}}(0) = 43\,\mathrm{\mu m\,s^{-1}}$ and $\alpha = 0.019\,\mathrm{\mu m\,s^{-2}}$. The initial conditions for both simulations are a quiescent fluid and a concentration profile of algae identical to the experimental one at $t = 0$ when the blue LED is switched on.

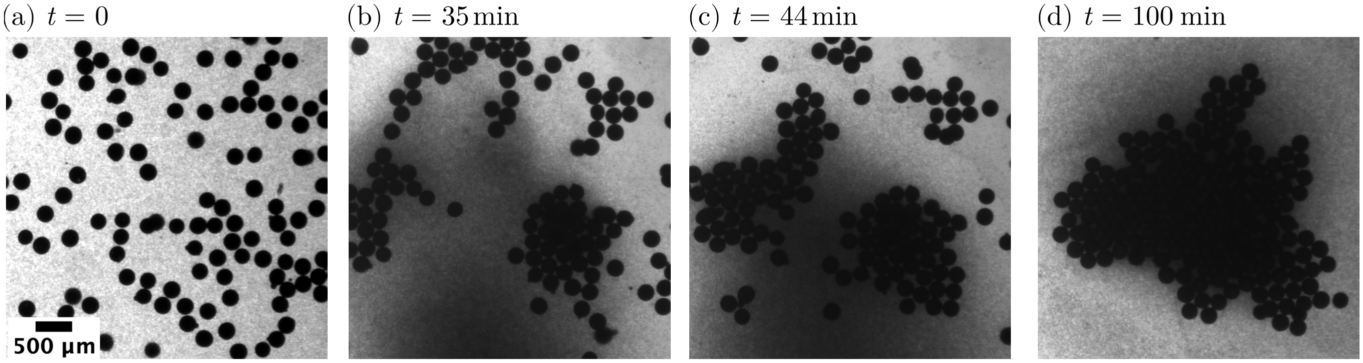

Figure S14. **Raft formation**. Time-lapse of an experiment with $d_b = 230\,\mu\text{m}$ beads in a *C. reinhardtii* suspension at $\text{OD}_i = 10$. Initially, both beads and algae are homogeneously distributed. Both side LEDs are switched on, creating a concentrated algae region far from the walls. Buoyant beads are drawn into the convection rolls and accumulate, forming a large raft of 158 beads around the plume. (a) $t = 0$. (b) $t = 35\,\text{min}$. (c) $t = 44\,\text{min}$. (d) $t = 100\,\text{min}$. See Movie S6.

away from the light at a constant swimming speed u_{photo}, i.e. without any light adaptation, do a good job in capturing the shape of the algal concentration profile when an equilibrium is reached, see Figure S13. However, the slow time evolution at long times $t \geq 10\,\text{min}$, which results in a flattening of the experimental profile, is not found in the simulations. By adding a time-dependent component to the swimming speed, such as $u_{\text{photo}}(t) = u_{\text{photo}}(0) - \alpha t$, we are able to properly capture both the initial accumulation of algae and its flattening at long times (see Figure S13c).

D. Raft formation

To illustrate the potential for medium cleaning, we place floating 230 μm beads in a dense *C. reinhardtii* suspension at $\text{OD}_i = 10$. Both lateral LEDs were switched on with different intensities, creating a highly concentrated algae plume near the left wall, see Figure S14. This plume exerts a strong attraction on buoyant beads, typically those near the top lid. As convection rolls develop, the plume captures these lighter beads, eventually forming a large aggregate of more than 150 beads, over the span of an hour and a half, measuring around 3-4 mm in length, see Figure S14d.

[1] J.-Y. Tinevez, N. Perry, J. Schindelin, G. M. Hoopes, G. D. Reynolds, E. Laplantine, S. Y. Bednarek, S. L. Shorte, and K. W. Eliceiri, "Trackmate: An open and extensible platform for single-particle tracking," *Methods*, vol. 115, pp. 80–90, 2017.

[2] M. Polin, I. Tuval, K. Drescher, and R. E. Goldstein, "Chlamydomonas swims with two "gears" in a eukaryotic version of run-and-tumble locomotion," *Science*, vol. 325, no. 5939, pp. 487–490, 2009.

[3] J. Arrieta, A. Barreira, M. Chioccioli, M. Polin, and I. Tuval, "Phototaxis beyond turning: persistent accumulation and response acclimation of the microalga chlamydomonas reinhardtii," *Scientific Reports*, vol. 7, no. 1, p. 3447, 2017.

[4] J. Dervaux, M. Capellazzi Resta, and P. Brunet, "Light-controlled flows in active fluids," *Nature Physics*, vol. 13, no. 3, pp. 306–312, 2017.

[5] J. Arrieta, M. Polin, R. Saleta-Piersanti, and I. Tuval, "Light control of localized photobioconvection," *Physical Review Letters*, vol. 123, no. 15, p. 158101, 2019.

[6] A. A. Fragkopoulos, F. Böhme, N. Drewes, and O. Bäumchen, "Metabolic activity controls the emergence of coherent flows in microbial suspensions," *Proceedings of the National Academy of Sciences*, vol. 122, no. 4, p. e2413340122, 2025.

[7] Z. Wang, S. A. Bentley, J. Li, K. Y. Wan, and A. C. Tsang, "Light-dependent switching of circling handedness in microswimmer navigation," *bioRxiv*, pp. 2025–08, 2025.

[8] M. A. Bees, "Advances in bioconvection," *Annual Review of Fluid Mechanics*, vol. 52, pp. 449–476, 2020.

[9] A. Mayer, "Chlamydomonas: Adaptation phenomena in phototaxis," *Nature*, vol. 217, no. 5131, pp. 875–876, 1968.
